# The Discovery of the Fucoidan-Active Endo-1→4-α-L-Fucanase of the GH168 Family, Which Produces Fucoidan Derivatives with Regular Sulfation and Anticoagulant Activity

**DOI:** 10.3390/ijms25010218

**Published:** 2023-12-22

**Authors:** Artem S. Silchenko, Ilya V. Taran, Roza V. Usoltseva, Nikolay V. Zvyagintsev, Anastasiya O. Zueva, Nikita K. Rubtsov, Dana E. Lembikova, Olga I. Nedashkovskaya, Mikhail I. Kusaykin, Marina P. Isaeva, Svetlana P. Ermakova

**Affiliations:** 1 Laboratory of Enzyme Chemistry, G.B. Elyakov Pacific Institute of Bioorganic Chemistry, Far-Eastern Branch of the Russian Academy of Sciences, 159, Prospect 100-Let Vladivostoku, 690022 Vladivostok, Russiausoltseva-r@yandex.ru (R.V.U.); zstasya95@gmail.com (A.O.Z.); rubtsov.nk@yandex.ru (N.K.R.); dlembikova@gmail.com (D.E.L.); mik@piboc.dvo.ru (M.I.K.); 2 Laboratory of Physical and Chemical Research Methods, G.B. Elyakov Pacific Institute of Bioorganic Chemistry, Far-Eastern Branch of the Russian Academy of Sciences, 159, Prospect 100-Let Vladivostoku, 690022 Vladivostok, Russia; 3 Laboratory of Microbiology, G.B. Elyakov Pacific Institute of Bioorganic Chemistry, Far-Eastern Branch of the Russian Academy of Sciences, 159, Prospect 100-Let Vladivostoku, 690022 Vladivostok, Russia; olganedashkovska@piboc.dvo.ru; 4 Laboratory of Marine Biochemistry, G.B. Elyakov Pacific Institute of Bioorganic Chemistry, Far-Eastern Branch of the Russian Academy of Sciences, 159, Prospect 100-Let Vladivostoku, 690022 Vladivostok, Russia; issaeva@gmail.com

**Keywords:** GH168 family, fucoidan-degrading gene cluster, recombinant fucanase, fucoidanase, *Fucus evanescens*, fucoidan derivatives, sulfation pattern, anticoagulant activity

## Abstract

Sulfated polysaccharides of brown algae, fucoidans, are known for their anticoagulant properties, similar to animal heparin. Their complex and irregular structure is the main bottleneck in standardization and in defining the relationship between their structure and bioactivity. Fucoidan-active enzymes can be effective tools to overcome these problems. In the present work, we identified the gene *fwf5* encoding the fucoidan-active endo-fucanase of the GH168 family in the marine bacterium *Wenyingzhuangia fucanilytica* CZ1127^T^. The biochemical characteristics of the recombinant fucanase FWf5 were investigated. Fucanase FWf5 was shown to catalyze the endo-type cleavage of the 1→4-O-glycosidic linkages between 2-O-sulfated α-L-fucose residues in fucoidans composed of the alternating 1→3- and 1→4-linked residues of sulfated α-L-fucose. This is the first report on the endo-1→4-α-L-fucanases (EC 3.2.1.212) of the GH168 family. The endo-fucanase FWf5 was used to selectively produce high- and low-molecular-weight fucoidan derivatives containing either regular alternating 2-O- and 2,4-di-O-sulfation or regular 2-O-sulfation. The polymeric 2,4-di-O-sulfated fucoidan derivative was shown to have significantly greater in vitro anticoagulant properties than 2-O-sulfated derivatives. The results have demonstrated a new type specificity among fucanases of the GH168 family and the prospects of using such enzymes to obtain standard fucoidan preparations with regular sulfation and high anticoagulant properties.

## 1. Introduction

Fucoidans are fucose-rich, sulfated polysaccharides produced by diverse species of brown algae. The structure of these polysaccharides can vary significantly in terms of the monosaccharide composition (sulfated fucans, sulfated galactofucans, sulfated fucogalactans, etc.), types of α- and β-glycosidic bonds between monosaccharide residues, and substituents (mainly sulfation, acetylation, and/or branching) [1,2,3]. Fucoidan-related fucose-containing polysaccharides were found in the jelly coat of sea urchin eggs (sulfated fucans) [4], the body walls of holothurians (sulfated fucans and fucosylated chondroitin sulfates) [5], as well as some genera of diatoms [6]. These polysaccharides have garnered considerable attention due to their diverse range of biological activities, including but not limited to antiviral, anticancer, antioxidant, radiosensitizing, radioprotective, antithrombotic, anticoagulant, and other activities [2,3,7,8,9]. The complexity and heterogeneity of such biopolymers are the main obstacles to research into the relationship between their structure and biological activity and to the use of these polysaccharides for various biomedical applications [10].

Fucoidan-active enzymes are promising tools for the targeted modification of fucoidan molecules to establish the relationship between fucoidan’s structures and their biological effects [11,12]. Sources of fucoidan-active enzymes may arise from diverse marine bacteria that utilize sulfated fucose-containing polysaccharides as a nutritional source. Such bacteria typically produce a large number of putative fucoidan-active enzymes, but many of them still have unknown functions [13]. At present, several fucoidan-active α-L-fucosidases (cleavage of terminal L-fucose residues in fucoidans and/or fucooligosaccharides) [14,15], sulfatases (elimination of sulfate groups from sulfated L-fucose residues in fucoidans and/or fucooligosaccharides) [16,17,18], and endo-fucanases (syn. fucoidanases or fucoidan hydrolases) have been described [19,20,21,22,23,24,25,26,27,28,29,30].

Endo-fucanases are involved in the depolymerization of fucoidans or sulfated fucans by cleavage of the glycosidic bonds between sulfated L-fucose residues via a hydrolytic mechanism [12]. According to the CAZY (Carbohydrate-active Enzymes) database, endo-fucanase activity is described for the glycoside hydrolase (GH) families 107, 168, and 174 (www.cazy.org (accessed on 1 August 2023)). Endo-fucanases of the GH107 family are the most studied. The known members of the GH107 family of enzymes catalyze the hydrolysis of either α-1→4 (EC 3.2.1.212) or α-1→3-glycosidic bonds (EC 3.2.1.211) between sulfated L-fucose residues in fucoidans. In addition to being specific for certain types of glycosidic linkages, these enzymes are also specific for certain sulfation patterns in fucoidans [21,22]. For example, α-(1→4)-glycosidic bonds between 2-O-sulfated L-fucose residues are cleaved by endo-fucanases FFA2, Fhf1, and Fhf2 [23,24,28], whereas the endo-fucanases FcnA, FFA1, and FWf4 can cleave fucoidans containing alternating 2-O- and 2,3-di-O-sulfated L-fucose residues [19,22,27]. Much less is currently known about the endo-fucanases of the GH168 and GH174 families. Only one representative of each family, GH168 and GH174, has been characterized in detail [20,31]. Both enzymes, FunA (GH168 family) and Fun174A (GH174 family), were shown to catalyze 1→3-glycosidic bond cleavage between 2-O-sulfated and non-sulfated L-fucose residues in sulfated fucans from sea cucumbers. Interestingly, neither FunA nor Fun174A showed any significant activity toward fucoidans isolated from different species of brown algae. Therefore, the diversity of specificities of the endo-fucanases of these families remains unexplored and requires further study.

The heparan sulfate proteoglycans, which are located on endothelial cells, play a significant role in the regulation of blood coagulation [32]. The utilization of heparin, which is a carbohydrate component of these proteoglycans, remains a prevalent practice in medicine as a potent anticoagulant and as a coating for some biomedical devices [33]. The anticoagulant effect of heparin is based on the selective allosteric activation of antithrombin III and/or heparin cofactor II, which are specific inhibitors of the serine proteases such as thrombin (factor IIa) and Xa factor. Inhibition of these proteases leads to inactivation of the coagulation cascade [34,35]. Animal tissues are the main source of heparin, which affects the cost, ecology, and risk of dangerous contaminations [36]. These factors are driving researchers to develop new anticoagulants [33].

Fucoidans and other sulfated fucose-containing polysaccharides of marine origin are known to have anticoagulant properties similar to heparin [9,37,38,39,40]. The plant nature and the high content of fucoidans in brown algae make them relatively cheap, safe, and environmentally friendly anticoagulants. However, not all fucoidans and/or their derivatives showed anticoagulant activity [41,42,43]. The monosaccharide composition, molecular weight, number, and position of the sulfate groups are thought to be important determinants of the anticoagulant activity of fucoidans [37,38]. However, as mentioned above, the macro- and microheterogeneity of these polysaccharides is the main bottleneck for standardization and reliable identification of the structural elements responsible for their anticoagulant activity.

In the present work, a bioinformatic, biochemical, and functional analysis of the novel recombinantly expressed fucoidan-active endo-fucanase FWf5 of the GH168 family from the marine bacterium *Wenyingzhuangia fucanilytica* CZ1127^T^ was performed. Using a series of sulfated polysaccharides and sulfated fucooligosaccharides with defined structures in combination with nuclear magnetic resonance (NMR) spectroscopy, the detailed specificity of this enzyme was reliably determined. The prospects for the application of this enzyme for the structural studies of fucoidans and for the production of defined sulfation-rich fucoidan derivatives have been demonstrated. Enzymatic fucoidan derivatives of different structures were obtained and further modified with the specific endo-4-O-sulfatase SWF5 and used to identify the structural determinants of the fucoidan responsible for its anticoagulant activity. It has been found that the high molecular weight and the 2,4-di-O-sulfation make a significant contribution to the anticoagulant activity of the fucoidan from brown algae *Fucus evanescens*. The endo-fucanase FWf5 of the GH168 family has been shown to produce a fucoidan derivative with the regular alternating 2-O- and 2,4-di-O-sulfation and anticoagulant activity similar to that of low-molecular-weight heparin (enoxaparin) and a native fucoidan with irregular structure. The enzyme obtained can, therefore, be used to produce a preparation of the structurally standardized fucoidan with high anticoagulant properties.

## 2. Results and Discussion

**Figure 1:**
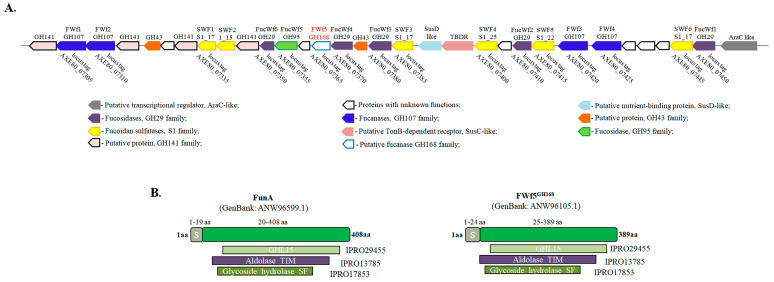
Fucoidan-degrading gene cluster of the marine bacterium *W. fucanilytica* CZ1127^T^. Locus tags are indicated for genes encoding enzymes that were previously recombinantly expressed and/or characterized (**A**). The gene encoding the putative fucanase FWf5 of the GH168 family is highlighted in red text. Domain organization of the fucanase FWf5 and the previously characterized endo-fucanase FunA (**B**).

The marine bacterium *W. fucanilytica* CZ1127^T^ has demonstrated high potential for the degradation of fucoidans of brown algae and sulfated fucans of echinoderms [21,44,45]. We previously identified a gene cluster of *W. fucanilytica* CZ1127^T^ that is potentially involved in the degradation of fucoidans of brown algae (Figure 1) [18]. Further detailed analysis of this cluster showed that the genes encode numerous glycoside hydrolases from various families, including endo-fucanases of the GH107 family, fucosidases of the GH29 and GH95 families, and GH168 family. We have named a putative enzyme of the GH168 family as FWf5 (AXE80_07365, GenBank accession number: ANW96105.1). Previously, it was demonstrated that some GH168 family members display endo-fucanase activity against sulfated fucans of echinoderms but are incompetent against fucoidans of brown algae [20]. We hypothesized that the occurrence of the gene encoding the putative GH168 enzyme in the fucoidan-degrading gene cluster denotes its involvement in the degradation of brown algae’s fucoidans.

### 2.1. Amino Acid Sequence Analysis of FWf5 

Bacterial glycoside hydrolases often display a modular (syn. domain) organization of their polypeptide chains [46]. Such domains can perform a variety of auxiliary functions that may enhance the binding to the substrate, define the sub-cellular localization of the enzyme in bacteria, or maintain the correct orientation of the catalytic domain [46,47,48]. For example, some endo-fucanases of the GH107 family contained 3–4 additional domains in addition to the catalytic domain [21,30]. We analyzed the amino acid sequence of FWf5 for the presence of such domains. The FWf5, like the previously described endo-fucanase FunA of the GH168 family, contained only the catalytic domain and the signal sequence (Figure 1 and Figure S2). The amino acid sequences of FWf5 and putative enzymes of the GH168 family deposited in the CAZy database (August 2023) showed different identity values ranging from 21 to 71%. The sequence identity of FWf5 and FunA is 57% (Table S2).

**Figure 2:**
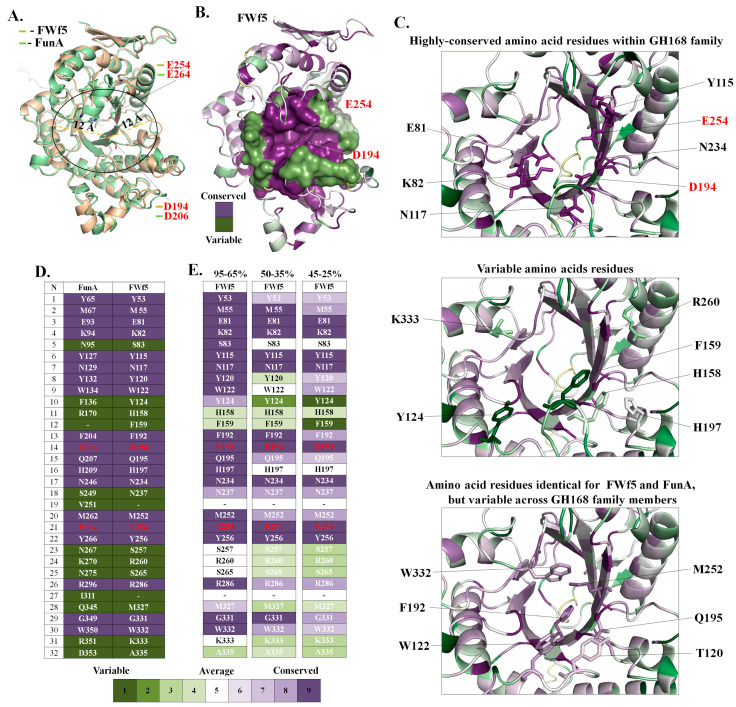
Structural analysis of the 3D model of FWf5. (**A**) Structural alignment of the AlphaFold predicted 3D models of the characterized endo-1→3-α-L-fucanaseFunA and FWf5. The 12Å regions around the catalytic residues D206 in FunA and D196 in FWf5 were selected for comparative analysis of the putative active sites of the GH168 fucanases. (**B**) Proposed model of FWf5. The putative active site is shown as surface-colored pairwise differences between FWf5 and FunA. (**C**) Relevant portion of the putative active site of FWf5 showing the conservation and location of some key amino acid residues. (**D**) Table showing pairwise comparison of the composition of solvent-accessible amino acid residues in the putative active sites of FWf5 and FunA. (**E**) Tables showing the conservation of amino acid residues in the active site of FWf5 among members of the GH168 family with different percentage identities to FWf5 ranging from 95 to 65%, from 50 to 35%, or from 45 to 25%. The catalytic amino acids of the FWf5 and FunA active sites are highlighted in red.

We performed a comparative analysis of the conserved amino acid residues of the putative active sites of FWf5 and FunA. Since data on the spatial structures of the GH168 family enzymes based on X-ray diffraction data are not yet available, we used data based on the AlphaFold prediction algorithm (UniProt accession: A0A1B1Y5R0). The AlphaFold has shown a high degree of accuracy in predicting protein folds in most cases [49]. The residues D206 and E264 have previously been shown to be essential for the function of fucanase FunA [20]. Their mutual arrangement in the predicted FunA model suggests that they are likely to act as an acid/base and nucleophile pair during the hydrolysis of glycosidic bonds (Figure 2A). The corresponding residues in the FWf5 model are D196 and E254. We performed a comparative analysis of the composition and arrangement of solvent-accessible amino acid residues within 12Å of D206 and D196 in the FunA and FWf5 models, respectively. These selected residues potentially form the putative cavities of the FunA and FWf5 active sites (Figure 2B). The analysis shows that the composition and location of more than half of the amino acid residues in the active sites of FWf5 and FunA are identical (Figure 2B,D). Significant differences between the structures of FWf5 and FunA are observed in the loop and disordered regions surrounding their active site cavities (Figure 2B).

ConSurf analysis of the FWf5 amino acid sequence using its homologs with identity ranges of 45–25%, 50–35%, and 95–65% allowed us to determine the conservation of amino acid residues in the proposed FWf5 active site between close and distant homologs (Figure 2E). The analysis showed that the residues E81, K82, Y115, N117, D194, N234, and E254 are highly conserved even in distant homologs of FWf5 (Figure 2B,E). Apparently, this group of amino acid residues is essential for the catalytic activity of the glycoside hydrolases of the GH168 family. Some residues, such as Y124, F159, H158, H197, R260, and K333, are variable in most of the GH168 sequences. The occurrence of these residues in the GH168 amino acid sequences decreases as the degree of identity with FWf5 decreases (Figure 2C,E). These residues are also different in FWf5 and FunA (Figure 2C,D). This may indicate the importance of these residues for FWf5, and it is likely that they define the differences in specificity between FWf5, FunA, and other GH168 family members. The residues T120, W122, F192, Q195, M252, and W332 are identical in the sequences of FWf5 and FunA but are variable in those of many other members of the GH168 family (Figure 2C,D). This indicates some similarity between FWf5 and FunA and the importance of these residues for both enzymes. These residues are mainly represented by aromatic amino acids, which are often involved in the recognition and binding of certain carbohydrate residues through CH/π interactions [50]. Presumably, these amino acid residues may be involved in the binding and proper accommodation of the fucosyl residues of substrates. It should be noted that the data obtained are based on bioinformatic predictions and require further confirmation by X-ray diffraction analysis.

### 2.2. Expression, Isolation, and Biochemical Properties of FWf5

In order to determine the putative function of FWf5, we produced this enzyme without the predicted signal peptide of 24 amino acids length. The target protein was purified from the *Escherichia coli* Arctic Express (DE3) cell lysate using single-step Ni-IMAC chromatography. The molecular mass of the protein obtained was 44.3 ± 1 kDa according to SDS-PAGE, which is close to the calculated molecular mass of this protein, 44.9 kDa (Figure S3).

We have investigated some biochemical properties of the fucanase FWf5 using fucoidan FeF from *Fucus evanescens* as substrate. The fucanase activity was analyzed by using the C-PAGE method. The presence of fucanase activity was detected by the appearance of bands of sulfated oligosaccharides, products of fucoidan depolymerization, in the electropherogram. Other methods based on the detection of reducing sugars, such as the Somogyi–Nelson or BCA methods [51,52], have been unsuccessful. The inability to detect fucanase activity with the conventional reducing sugar detection methods has been previously described for some GH107 family endo-fucanases [19].

**Figure 3:**
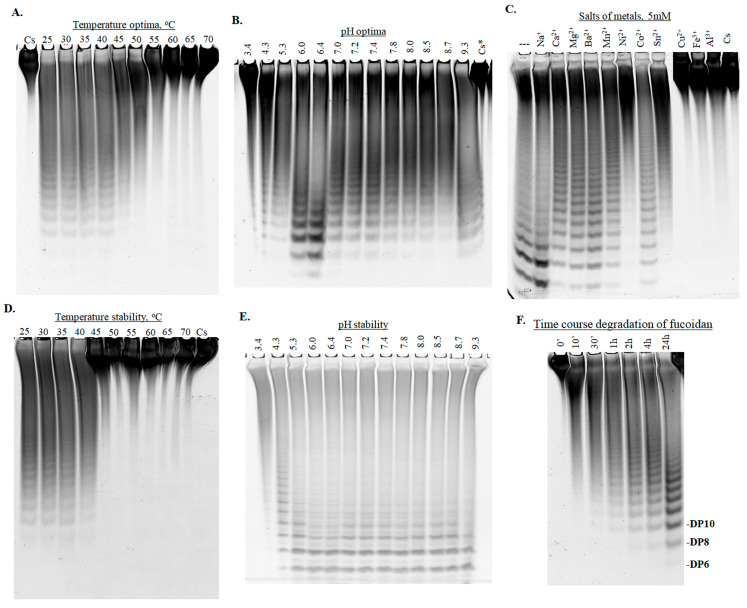
Electropherogram of the hydrolysis products of fucoidan FeF produced by FWf5 under different reaction conditions. Effect of buffers with different pH (**A**), temperatures (**B**), and metal cations (**C**) on the enzymatic activity of FWf5. Stability of FWf5 in buffers with different pH (**D**) and temperatures (**E**). (---) FWf5 without the addition of metal cations. Cs is the non-hydrolyzed fucoidan FeF. Cs* is the non-hydrolyzed fucoidan FeF in Na-succinate buffer pH 3.4. (**F**) Time course of degradation of the fucoidan FeF by FWf5. DP6, DP8, and DP10 are the expected degrees of polymerization of oligosaccharides corresponding to the electrophoretic mobility of 2O-sulfated fuco-hexa-, fuco-octa- and fuco-decasaccharides.

The pH range of 6.0–6.4 and temperatures of 25 to 40 °C were optimal for the enzymatic activity of the fucanase FWf5 (Figure 3A,B). The pH optimum differed significantly from that determined for FunA, which had a maximum pH of 8.0 [20]. The fucanase lost enzymatic activity when incubated at temperatures above 40 °C for 20 min (Figure 3D). The FWf5 was pH tolerant and remained stable at pH intervals from 5.3 to 8.7 (Figure 3E). The temperature and pH stability values for FWf5 are close to those determined for the endo-fucanase FunA.

Metal ions can modulate the activity of some endo-fucanases. Some enzymes of the GH107 family do not show enzymatic activity without certain metal ions [21,22]. The fucanase FWf5 catalyzed the hydrolysis of fucoidan FeF without additional metal ions (Figure 3C). Ions Cu^2+^, Fe^3+^, and Al^3+^ completely inhibited the activity of FWf5. Ions Ca^2+^, Mg^2+^, Ba^2+^, Mn^2+^, and Co^2+^ reduced the activity of FWf5.

The cleavage of fucoidan FeF by FWf5 proceeded with a gradual accumulation of oligosaccharides with different degrees of polymerization (Figure 3F). This indicates the endo-type action of this enzyme. No accumulation of a dominant reaction product during fucoidan hydrolysis was detected. The FWf5 is, therefore, not a processive enzyme and cleaves fucoidan FeF in a random manner. A similar observation was made for the previously characterized fucanase FunA [20].

### 2.3. Comparison of the Effects of Some Members of the GH168 Family on the Hydrolysis of Marine Sulfated Polysaccharides

Some members of the GH168 family have been reported to be inactive against both sulfated fucans of echinoderm and fucoidans of brown algae [20]. It is possible that the substrates for some of the GH168 family enzymes are poly- and/or oligosaccharides consisting of monosaccharides other than L-fucose. Therefore, the search for novel activities among the GH168 family members may require a wide range of substrates with different structures.

**Figure 4:**
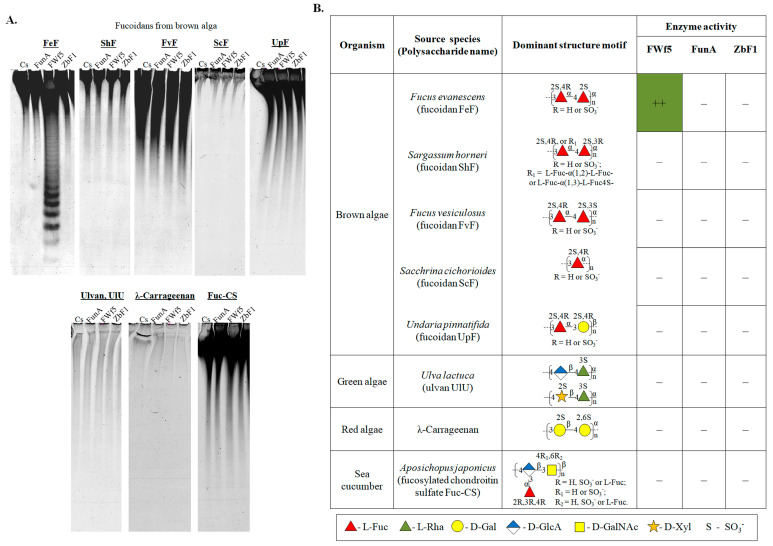
Analysis of the polysaccharide degrading activity of the FWf5, ZbF1, and FunA of the GH168 family against sulfated polysaccharides of different structures. (**A**) Electropherograms of the hydrolysis products of sulfated polysaccharides produced by FWf5 and FunA. Cs refers to the non-hydrolyzed sulfated polysaccharide. (**B**) The table illustrating the effects of FWf5 on sulfated polysaccharides. The data on enzymatic activity were derived from C-PAGE analysis (**A**). Fucoidanase activity was not detected (−), and high activity was indicated by (++) notations. Carbohydrate residues are depicted according to the symbol nomenclature for glycans as outlined in [54].

We compared the ability of FWf5, endo-1→3-α-L-fucanaseFunA, and putative GH168 enzyme ZbF1 to catalyze the hydrolysis of sulfated polysaccharides isolated from different marine sources such as brown algae (fucoidans), green algae (ulvans), red algae (carrageenans), and sea cucumber (fucosylated chondroitin sulfate) (Figure 4A,B). The structural characteristics of fucoidans used in the experiment are shown in Table S1. The gene for ZbF1 has been determined to be part of the polysaccharide utilization locus (PUL) of the marine bacterium *Zobellia barbeyronii* KMM 6746^T^ (see pages 2–3 of the Supplementary Materials), potentially involved in degrading fucose-containing sulfated polysaccharides or ulvans.

The endo-1→3-α-L-fucanase FunA showed no activity towards fucoidans isolated from various brown algae used in the experiment (Figure 4A). This enzyme has previously been shown to cleave internal 1→3-O-glycosidic linkages between 2-O-sulfated and non-sulfated L-fucose residues in a sulfated fucan from *Isostichopus badionotus* whose backbone is composed of 1→3-O-linked sulfated L-fucose residues [20]. The lack of effect of FunA on fucoidan ScF from *Saccharina cichorioides* (former name *Laminaria cichorioides*), which has a similar backbone structure, can be caused by the absence or low content of fragments that the enzyme can cleave. Fucoidan from the brown alga *S. cichorioides*, in contrast to the sulfated fucan from *I. badionotus*, is predominantly sulfated at C2 and C2/C4 of the L-fucose residues (Figure 4B) [53]. This indicates that this fucanase is highly selective for the location of sulfate groups on L-fucose residues in potential substrates.

Similar to FunA, fucanase FWf5 did not catalyze the hydrolysis of most of the fucoidans used in the experiment, such as ScF from *S. cichorioides*, UpF from *Undaria pinnatifida* [55], ShF from *Sargassum horneri,* or FvF from *F. vesiculosus* (Figure 4A,B). Low-molecular-weight products of enzymatic hydrolysis were only detected after treatment of the fucoidan FeF from *F. evanescens* by FWf5. It should be noted that fucoidans isolated from the brown algae *F. evanescens* (FeF), *F. vesiculosus* (FvF), and *S. horneri* (ShF) have a similar backbone structure, consisting mainly of alternating 1→3-; 1→4-linked α-L-fucose residues, but have different sulfation patterns [56,57,58]. The lack of enzymatic activity against fucoidans from *F. vesiculosus* and *S. horneri* highlights the importance of the position of sulfate groups at L-fucose residues for the FWf5 to hydrolyze glycosidic bonds.

As expected, neither FunA nor FWf5 are able to catalyze the degradation of λ-carrageenan, sulfated xylo-glucurono-ramnan UlU (ulvan) from *Ulva lactuca,* and fucosylated chondroitin sulfate Fuc-CS from *Apostichopus japonicas* (Figure 4A,B). The data obtained show that FunA and FWf5 have a narrow specificity for certain structures of fucoidans and/or sulfated fucans. These enzymes are not catalytically promiscuous towards sulfated polysaccharides whose structure differs from the target.

No reaction products were detected after the action of the ZbF1 of the GH168 family on the polysaccharides used in the experiment (Figure 4A,B). The data obtained indicate that the ZbF1 is unable to catalyze cleavage within the structural fragments of fucoidans, carrageenans, or ulvans shown in Figure 4B. However, the structural diversity of fucoidans, carrageenans, and ulvans is not limited to the substrates used in the experiment. Therefore, the functional activity of ZbF1 remains unclear and needs to be further investigated using a broader set of substrates.

**Figure 5:**
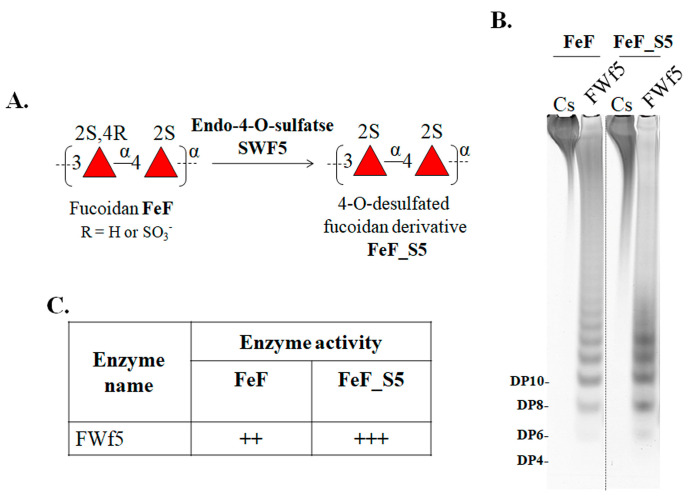
Effect of FWf5 on the fucoidan FeF and its 4-O-desulfated derivative FeF_S5. (**A**) Scheme for obtaining the 4-O-desulfated derivative FeF_S5 from fucoidan FeF using the fucoidan endo-4-O-sulfatase SWF5. (**B**) Electropherogram of the fucoidan FeF and its derivative FeF_S5 hydrolysis products produced after by FWf5. (**C**) The table of fucanase activity of FWf5 against FeF and its’ 4-O-desulfated derivative. (++) Middle activity; (+++) high activity.

The role of a specific position of sulfate groups in the fucoidan FeF on the efficiency of its hydrolysis by the fucanase FWf5 was further investigated. Fucoidan from *F. evanescens* has previously been shown to consist of the alternating 1→3- and 1→4-linked L-fucose residues, which are mainly sulfated at C2 and C2/C4 (Figure 4B) [56,59]. We have removed the 4-O-sulfation in fucoidan FeF using the specific endo-4-O-sulfatase SWf5, resulting in the 4-O-desulfated derivative FeF_S5 with the dominant structure of [→3-α-L-Fucp2S-1→4-α-L-Fucp2S-1→]_n_ (Figure 5A). It is shown that after treatment of the derivative FeF_S5 with the FWf5, the amount of oligosaccharides released was higher than after treatment of the native fucoidan FeF. Therefore, the removal of 4-O-sulfation in fucoidan increases the amount of glycosidic linkages available for hydrolysis by FWf5, indicating the selectivity of FWf5 towards 2-O-sulfation in a (1→3;1→4)-α-L-fucoidans.

The data obtained show that the fucanases of the GH168 family can exhibit different specificities with respect to the type of glycosidic linkages, α-1→3 or alternating α-1→3;1→4, present in the fucoidan or sulfated fucan backbones. Moreover, the specific location of the sulfate groups in the substrate molecules appears to be necessary for the cleavage of the glycosidic linkages by the fucanases FWf5 and FunA. Similar differences in specificity towards the type of glycosidic linkages and certain sulfation patterns have been shown for fucanases belonging to the GH107 family [21,22,24].

A study of the activity of three members of the GH168 family enzymes against structurally different polysaccharides showed that the enzymes studied apparently have different specificities. As shown, even small differences in the structure of the substrates can result in the inability to detect the activity of this family of enzymes. This emphasizes the narrow specificity of representatives of this family. A large set of substrates with a defined structure may be required to search for the functional activity of some members of the GH168 family.

### 2.4. Evaluation of the Structure of Products of Enzymatic Hydrolysis of the Fucoidan from F. evanescens by FWf5

The information about the structure of the reaction products can provide comprehensive information about the specificity of the enzymes. Fucoidan FeF isolated from *F. evanescens* was exhaustively hydrolyzed by FWf5 over 72 h. Notably, although the FWf5 hydrolyzed the fucoidan, it did not entirely convert it to oligosaccharides. In addition to the formation of sulfated oligosaccharides, higher-molecular-weight fragments were also formed. A similar observation has been reported previously for some endo-fucanases of the GH107 family [23,28]. This is attributed to the strict specificity of the GH107 family enzymes. They are unable to cleave fucoidan fragments that do not match their specificity.

**Figure 6:**
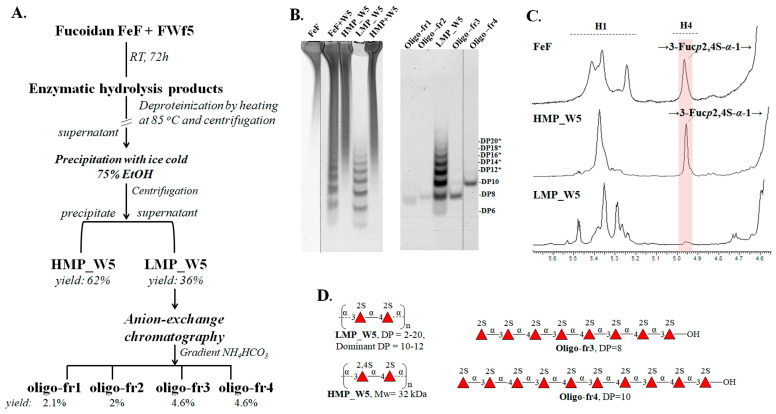
Hydrolysis of the fucoidan from *F. evanescens* by FWf5. (**A**) Scheme of preparation of high- (HMP_W5) and low-molecular-weight (LMP_W5) products of enzymatic hydrolysis and sulfated fucooligosaccharides. (**B**) C-PAGE analysis of fucoidan, fractions of hydrolysis products before treatment with 75% ethanol, and high- (HMP_W5) and low-molecular-weight (LMP_W5) fractions obtained after separation with 75% ethanol followed by centrifugation. HMP+W5 is the effect of the FWf5 enzyme on the HMP_W5 fraction. *—calculated degrees of polymerization of oligosaccharides. (**C**) The region of the ^1^H NMR spectra of the FeF, HMP_W5, and LMP_W5 showing H1 signals as well as signals corresponding to H4 of residues →3-α-L-Fucp2,4S-1→. (**D**) Schematic representation of the structures of the fractions LMP_W5, HMP_W5, and the oligosaccharides oligo-fr3 and oligo-fr4 obtained by separation of the LMP_W5 fraction.

To investigate the reasons for the limited depolymerization of fucoidan FeF by fucanase FWf5, we attempted to separate the enzymatic reaction products and determine their structures. Using 75% aqueous ethanol, we separated the low-molecular-weight enzymatic hydrolysis products (LMP_W5) from the high-molecular-weight products (HMP_W5) (Figure 6A). No oligosaccharide formation was detected when the HMP_W5 fraction was incubated with FWf5 (Figure 6B). This verifies that the HMP_W5 fraction is an end-point product resistant to further cleavage by the fucanase FWf5.

The LMP_W5 fraction was analyzed using C-PAGE and SEC methods (Figure 6B and Figure S4). The results indicated a distribution of oligosaccharides with a degree of polymerization (DP) ranging from 2 to 20 (Figure S4 and Figure 6B). The majority of oligosaccharides had a DP of 10 to 12 (Figure 6B). The SEC analysis showed that the average molecular weight of the obtained HMP_W5 fraction was about 32 kDa (Figure S5).

The ^1^H NMR spectra of the obtained fractions LMP_W5 and HMP_W5 were significantly different (Figure 6C). In the ^1^H spectra of the LMP_W5 fraction, there were no signals at 4.94 ppm corresponding to H4 of →3-α-L-Fucp2,4S-1→ residues. These signals, however, were present in the ^1^H NMR spectra of the FeF and the HMP_W5. Since the structure of the fucoidan FeF mainly consists of fragments with 2-O- and alternating 2-O- and 2,4-di-O-sulfation, it can be assumed that the LMP_W5 fraction mainly contains sulfation at C2. This, therefore, suggests a different distribution of 2-O- and 2,4-di-O-sulfation in low- and high-molecular-weight reaction products formed during the hydrolysis of fucoidan by the FWf5.

The structure of the HMP_W5 fraction was further established by NMR spectroscopy using various 1D and 2D techniques (^1^H, ^13^C, COSY, HSQC, HMBC, and ROESY). The NMR spectra of the HMP_W5 fraction obtained were identical to the previously deciphered spectra of high-molecular-weight reaction products obtained with the endo-1→4-α-L-fucanases FFA2 or FWf4 of the GH107 family [22,28]. Thus, the HMP_W5 fraction is a polysaccharide with a regular structure [→4-α-L-Fucp2S-1→3-α-L-Fucp2,4S-1→]_n_ (Figure 6D). The chemical shifts of the HMP_W5 fraction are provided in Table 1.

To determine the specificity of FWf5 for the type of glycosidic bonds cleaved in fucoidan, sulfated oligosaccharides present in the fraction LMP_W5 were separated by anion-exchange chromatography (Figure 6A). As a result, several fractions, oligo-fr1, oligo-fr2, oligo-fr3, and oligo-fr4, were obtained. The oligo-fr3 and oligo-fr4 fractions contained mostly homogeneous oligosaccharides according to C-PAGE analysis (Figure 6B). Yields of the oligo-fr3 and oligo-fr4 fractions were 4.6% (4.6 mg) and 4.6% (4.6 mg), respectively. Analysis of the NMR spectra of the oligo-fr3 and oligo-fr4 fractions showed that they had mostly identical signals corresponding to their protons and carbons, indicating their structure similarity. The chemical shifts of the oligo-fr3 and oligo-fr4 oligosaccharides are shown in Table 2. Identical spectra to those of oligo-fr3 and oligo-fr4 were previously obtained for some oligosaccharides produced by the endo-fucanases FWf2 and Ffh1 of the GH107 family [21,24]. Thus, oligo-fr3 and oligo-fr3 are 2O-sulfated octa- and decasaccharides with the following structures α-L-Fucp2S-1→[3-α-L-Fucp2S-1→4-α-L-Fucp2S]_3_→3-α-L-Fucp2S and α-L-Fucp2S-1→[3-α-L-Fucp2S-1→4-α-L-Fucp2S-1]_4_→3-α-L-Fucp2S, respectively (Figure 6D).

The order of the glycosidic linkages 1→3-1→4-//-1→3 in the resulting oligo-fr3 and oligo-fr4 oligosaccharides indicates the cleavage of the 1→4-O-glycosidic linkages by the FWf5 in fucoidan FeF. Thus, the FWf5 is an endo-1→4-α-L-fucanase (EC 3.2.1.212) specific for 2-O-sulfated fragments in (1→3;1→4)-fucoidans. To our knowledge, this is the first characterized member of the GH168 family of endo-fucanases specific for 1→4-glycosidic linkages between sulfated L-fucose residues. Interestingly, the endo-fucanase FWf2 (locus tag AXE80_07310) of the GH107 family, encoded by the same fucoidan-degrading locus of the marine bacterium *W. fucanilytica* CZ1127^T^ (Figure 1), has a similar specificity [21].

### 2.5. Effect of FWf5 on Sulfated Fucooligosaccharides

**Figure 7:**
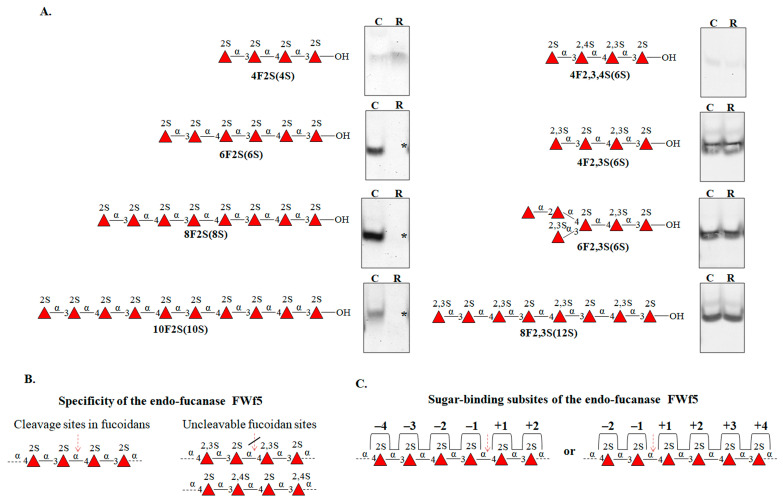
Substrate specificity of fucanase FWf5. (**A**) C-PAGE analysis of the effect of FWf5 on oligosaccharides of different structures. “C”—the control oligosaccharide without the addition of the FWf5. “R”—oligosaccharide incubated with the FWf5 for 24 h at RT. (*)—change in the electrophoretic mobility or disappearance of the oligosaccharide as a result of depolymerization. (**B**) The proposed sites in the fucoidans that are cleaved by the FWf5 and the structural motifs of the fucoidans that are resistant to the enzyme cleavage. (**C**) The proposed sugar-binding subsites of the FWf5, with subsites numbered according to the nomenclature [60].

The active sites of glycoside hydrolases are thought to consist of tandem sugar-binding subsites that recognize and accommodate specific structures of the substrate for further cleavage [60]. These subsites can vary in number, organization, and selectivity. The selectivity and topology of the sugar-binding subsites determine the specificity of the glycoside hydrolases and the degree of polymerization (DP) of the resulting reaction products. It was previously shown that the main product of the depolymerization of the sulfated fucan by the endo-fucanase FunA was a sulfated tetrasaccharide [20]. In contrast, the main products of the fucanase FWf5 were oligosaccharides with a degree of polymerization of 10–12 (Figure 7B). This may indicate a different number or topology of sugar-binding subsites in fucanases FunA and FWf5.

Oligosaccharides are convenient models for studying the specificity and topology of the sugar-binding subsites of O-glycoside hydrolases. We have tested the effect of the fucanase FWf5 on sulfated fucooligosaccharides with different structures. The FWf5 was shown to catalyze the cleavage of 2-O-sulfated fucooligosaccharides only (Figure 7A). The enzyme did not depolymerize oligosaccharides with additional 3-O or 4-O-sulfation (Figure 7A). This confirms the specificity of the fucanase for the cleavage of 1→4-O-glycosidic linkages between 2-O-sulfated α-L-fucose residues only. This enzyme cannot cleave 1→4-O-glycosidic linkages between 2-O- and 2,3-di-O- or 2-O- and 2,4-di-O-sulfated α-L-fucose residues (Figure 7B).

**Figure 8:**
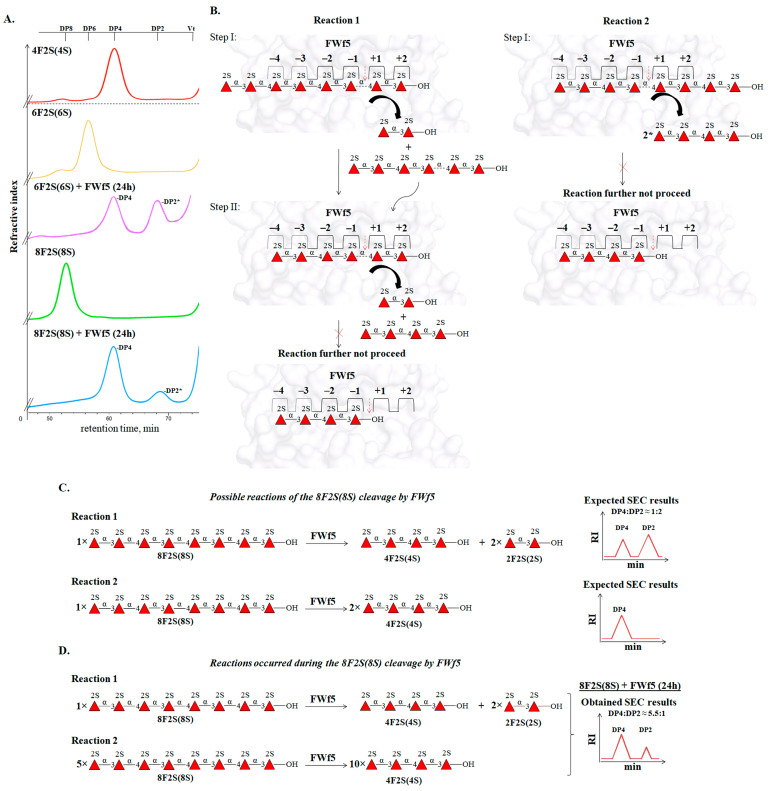
Analysis of the degradation of the oligosaccharides by endo-fucanase FWf5. (**A**) SEC analysis of the cleavage products of hexasaccharide 6F2S(8S) and octasaccharide 8F2S(8S) by the endo-fucanase during 24 h. (**B**) Proposed steps for the cleavage of the octasaccharide, taking into account its different position in the sugar-binding subsites of FWf5 (reaction 1 and reaction 2). (**C**) Diagrams of two possible reaction equations 1 and 2 of the octasaccharide 8F2S(8S) cleavage by FWf5. The expected results of the SEC analysis, indicating the ratio of tetra- (DP4) and disaccharides (DP2) in reaction 1 or 2, are indicated to the right of the reaction equations. (**D**) Experimentally obtained reaction equations 1 and 2 with stoichiometric coefficients. The stoichiometry of the reactions is based on the experimentally calculated ratio of DP4 and DP2 during 8F2S(8S) cleavage by FWf5 using SEC analysis, which is 5.5:1 (A, 8F2S(8S)+FWf5 (24 h)). Stoichiometric coefficients indicate a 5-fold dominance of reaction 2 over 1 during 8F2S(8S) cleavage by the FWf5.

The degree of polymerization of the substrate molecule is of great importance for the hydrolysis of glycosidic linkages by the FWf5. The enzyme did not cleave the 2-O-sulfated tetrasaccharide 4F2S(4S), whereas 2-O-sulfated hexa-, octa-, and decasaccharides were efficiently cleaved by this enzyme (Figure 7A). The ability to catalyze the hydrolysis of the hexasaccharide 6F2S(6S) suggests the presence of at least six significant sugar-binding substrates in the active site of FWf5 (Figure 7C). SEC analysis revealed that the end-point reaction products of the hexasaccharide cleavage by FWf5 were di- and tetrasaccharides (Figure 8A). Accordingly, the sugar-binding subsites in FWf5 have a topology of −4 and +2 or −2 and +4 (the numbering of the sugar-binding subsites follows the nomenclature [60]) (Figure 7C). Therefore, the FWf5 requires a minimum of six monosaccharide residues for substrate recognition and cleavage. Unfortunately, we were unable to determine the exact direction of the sugar-binding subsites of FWf5 because this enzyme did not catalyze the cleavage of the fluorescently labeled hexa- and octasaccharides we obtained. A similar effect on fluorescently labeled oligosaccharides was observed for the endo-fucanase FWf2 of the GH107 family [21].

The end products of the depolymerization of the 2-O-sulfated octasaccharide 8F2S(8S) by FWf5 were di- and tetrasaccharides (Figure 8A). This confirms the sugar-binding subsite topology of the FWf5 described above. However, SEC analysis of the reaction products revealed a tetra- to disaccharide ratio of 5.5 to 1. Such a ratio could not correspond to the occurrence of a single reaction due to the non-stoichiometric amount (Figure 8C). We hypothesized that this was due to two competing reactions, 1 and 2, in which the octasaccharide occupies different positions in the sugar-binding subsites of FWf5 (Figure 8B). According to the ratio of di- and tertasaccharides in the obtained reaction products, the preference of reaction 2 over reaction 1 differs about five times under the conditions used (Figure 8D). Since processive enzymes perform sequential steps of catalysis without dissociation of substrate molecules [61,62], a variant of reaction 1 or 2 may occur, but not both. This emphasizes that the FWf5 cleaves the polysaccharide in a random manner.

The data obtained indicate that the members of the GH168 family can be involved in the depolymerization of fucoidans of brown algae. As shown, the fucanase FWf5 is an endo-type enzyme that catalyzes the hydrolysis of 1→4-O-glycosidic linkages exclusively between the residues of 2-O-sulfated L-fucose in fucoidans composed of alternating 1→3- and 1→4-linked residues of sulfated L-fucose. The strict specificity of the enzyme for a particular sulfation in fucoidans and the structural features of the fucoidan from *F. evanescens* made it possible to obtain derivatives with regular sulfation.

### 2.6. Anticoagulant Activity of the Fucoidan FeF from F. evanescens and Its Enzymatic Derivatives

**Figure 9:**
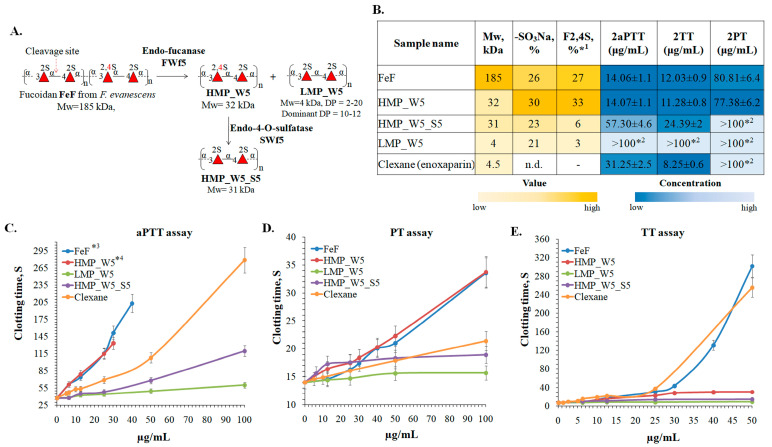
Scheme for the production of the derivatives HMP_W5, HMP_W5_S5, and LMP_W5 using the endo-1→4-α-L-fucanase FWf5 of the GH168 family and the endo-4-O-sulfatase SWF5 of the S1_22 subfamily (**A**). (**B**) Table of structural characteristics and 2aPTT, 2PT, and 2TT values of the fucoidan FeF and its enzymatically produced derivatives HMP_W5, HMP_W5_S5, and LMP_W5. Clexan—low-molecular-weight heparin (enoxaparin)—was used as positive control. *^1^—percent of the 2,4-di-O-sulfated L-fucopyranose residues present in fucoidan samples. It was calculated using ^1^H NMR (Figure S6). *^2^—the double-time blood coagulation in the TT and PT assay was not reached even at high concentrations (>100 μg/mL). Anticoagulant activity of the fucoidan FeF and its derivatives measured by aPTT (**C**), PT (**D**) or TT (**E**) assay. *^3^—aPTT values above 40 µg/mL are omitted as they exceeded the upper limit of quantitation (>1000s); *^4^—aPTT values above 30 µg/mL are omitted as they exceeded the upper limit of quantitation (>1000s).

Fucoidan from the brown alga *F. evanescent* has previously been shown to have potent anticoagulant properties in vitro and in vivo, but the structural elements responsible for this effect were not fully clear [63,64,65]. As shown, the use of the fucanase FWf5 to depolymerize fucoidan FeF resulted in the production of low- (LMP_W5) and high-molecular-weight (HMP_W5) derivatives with regular sulfation. To increase the structural diversity of the derivatives obtained, the HMP_W5 derivative was further 4-O-desulfated using the fucoidan endo-4-O-sulfatase SWF5. As a result, the 4-O-desulfated derivative HMP_W5_S5 with the following structure [→3-α-L-Fucp2S-1→4-α-L-Fucp2S-1→]_n_ and a molecular weight of 31 kDa was obtained. Some structural characteristics of fucoidan and the resulting enzymatic derivatives are shown in Figure 9A,B. Such derivatives can be used to reliably determine the relationship between the structure and biological effects of fucoidan from *F. evanescens*.

The effect of the fucoidan FeF and its derivatives HMP_W5, HMP_W5_S5, and LMP_W5 on blood clotting was evaluated. The anticoagulant activity was measured by the activated partial thromboplastin time (aPTT), prothrombin time (PT), and thrombin time (TT). The low-molecular-weight heparin Clexane (enoxaparin) was used as a positive control.

Significant differences in aPTT, PT, and TT levels were observed between obtained fucoidan samples (Figure 9B–E). Fucoidan FeF and its derivative HMP_W5 showed anticoagulant activity in the aPTT test higher than that of Clexane, while the derivative HMP_W5_S5 showed a moderate effect. The anticoagulant activity of the derivative LMP_W5 was insignificant in all aPTT, TT, and PT assays. Compared to Clexane, HMP_W5_S5, or LMP_W5, the fucoidan FeF and HMP_W5 showed a slight effect in the PT assay. All these differences are apparently caused by their structural differences, such as the molecular weight and the position of sulfate groups.

Molecular weight is considered to be an important element in the anticoagulant activity of fucoidans. A decrease in anticoagulant activity of low-molecular-weight fucoidan derivatives compared to native fucoidan has been reported in a number of studies [66,67,68,69]. Meanwhile, the reduced molecular weight fucoidan derivative obtained by photocatalytic degradation was reported to have similar activity to native fucoidan [70]. Our data confirm the negative effect of molecular weight reduction on the anticoagulant activity of fucoidans. Fucoidan FeF with a molecular weight of 185 kDa had a significantly higher anticoagulant activity than the derivatives HMP_W5_S5 and LMP_W5 with molecular weights of 31 kDa and 4 kDa, respectively (Figure 9B–E). The comparison of the aPTT values of the derivatives HMP_W5_S5 and LMP_W5 with identical structures [→3-α-L-Fucp2S-1→4-α-L-Fucp2S-1→]_n_ but different molecular weights showed a similar correlation (Figure 9C). The concentration that doubled the blood clotting time in the aPTT assay (2aPTT) was 57.3 μg/mL for the HMP_W5_S5, while it was over 100 μg/mL for the low-molecular-weight derivative LMP_W5 (Figure 9B). The data obtained reliably demonstrate that the high molecular weight makes a positive contribution to the anticoagulant effect of fucoidans.

Fucoidan FeF and the derivative HMP_W5 had almost identical 2aPTT, 2TT, and 2PT values despite a significant difference in molecular weight (Figure 9B–E). Apparently, the significant amount of 2,4-di-O-sulfation in the HMP_W5 compensated for the negative effect of the decrease in molecular weight. The effect of 2,4-di-O-sulfation is also confirmed by comparing the anticoagulant effects of the derivative HMP_W5 and its selectively 4-O-desulfated derivative HMP_W5_S5. The anticoagulant effect of the 2,4-di-O-sulfation-enriched derivative HMP_W5 was higher than that of Clexan and identical to FeF, but after 4-O-desulfation, the anticoagulant effect was either significantly reduced (aPPT assay) or almost lost (TT and PT assays) (Figure 9B–E). Our data reliably demonstrate that 2,4-di-O-sulfation plays a critical role in the anticoagulant activity of (1→3;1→4)-fucoidans. The importance of 2,4-di-O-sulfation for the anticoagulant activity of sulfated 3-linked fucans of echinoderms [71,72] and fucoidans of brown algae has been noted previously [69].

The inability of low-molecular-weight fucoidans to increase PT levels has been reported previously, suggesting a significant influence of molecular weight on this process [66,67]. We have also shown that the low-molecular-weight derivative LMP_W5 is not able to significantly affect PT levels. At the same time, the derivative HMP_W5_S5, with a molecular weight of 31 kDa and identical to the LMP_W5 structure, had a similar effect. However, the derivative HMP_W5, which has a comparable structure to the HMP_W5_S5 but with an additional 4-O-sulphation, showed a moderate impact in the PT test. Presumably, the ability of fucoidans to influence PT levels depends not only on the molecular weight but also on the specific location of the sulfate groups.

The data obtained show that fucoidan FeF and its enzymatic derivative HMP_W5 have anticoagulant properties higher than those of low-molecular-weight heparin (Clexane). The molecular weight and the sulfation pattern were the keys to the anticoagulant activity of the fucoidans. The presence of 2,4-di-O-sulfation leads to increased anticoagulant activity of fucoidan, whereas a decrease in molecular weight leads to a decrease in anticoagulant activity. Low-molecular-weight 2-O-sulfated derivatives have significantly reduced anticoagulant activity compared to the native fucoidan.

## 3. Materials and Methods

### 3.1. Reagents and Substrates

The strain of *W. fucanilytica* CZ1127^T^ was purchased from the Korean Collection for Type Cultures (KCTC 42864) (181 Ipsin-gil, Jeongeup-si, Jeollabuk-do, Korea) [45]. The strain of *Z. barbeyronii* KMM 6746^T^ was purchased from the Collection of Marine Microorganisms (KMM 6746) (G.B. Elyakov Pacific Institute of Bioorganic Chemistry, Vladivostok, Russia) [73].

The recombinant fucoidan endo-4-O-sulfatase SWF5 from the marine bacterium *W. fucanilytica* CZ1127^T^ was obtained as described in [17].

Brown algae *Fucus distichus* subsp. *evanescens* (further *F. evanescens*, August 2017) was collected at literal zone near Kunashir Island (Pacific Coast). Brown algae *Undaria pinnatifida* (July 2015) and *Saccharina cichorioides* (September 2015) were collected from the Troitsa Bay, Sea of Japan, Russian Far East, and *Sargassum horneri* (July 2016) were collected from the Huiquan bay, Yellow sea, Qingdao, China. Fucoidans from these types of algae were isolated and purified by the methods described or cited in reference [74]. Crude fucoidan from *Fucus vesiculosus* is commercial product of Sigma-Aldrich (Steinheim, Germany). Fucoidans isolated from *F. evanescens*, *F. vesiculosus,* and *U. pinnatifida*, which, according to the nuclear magnetic resonance (NMR) spectroscopy data, contained substantial amounts of acetate groups, were further O-deacetylated using the method described in [75]. The 4-O-desulfated polysaccharide derivative FeF_S5 was obtained using the endo-4-O-sulfatase SWF5 according to the method described in [17]. Green algae *U. lactuca* was collected from coast of Troitsa Bay, Sea of Japan, Russian Far East, in August 2022. Crude polysaccharide preparation was obtained from *U. lactuca* by the hot water method, as described in reference [76]. The resulting polysaccharides mixture was further fractionated by anion-exchange chromatography using DEAE-MacroPrep (Bio-Rad, Hercules, CA, USA) with a stepwise gradient of 0.5, 1, 1.5, and 2M NaCl water solutions. The fraction of the sulfated xylo-rhamno-glucuronan UlU that eluted with the 2M NaCl solution was used in the present work. Samples of sea cucumbers, *A. japonicas*, were collected in August 2021 from Troitsa Bay, Sea of Japan, Russian Far East, and fixed with 90% ethanol. Fucosylated chondroitin sulfate FUC-CS was isolated according to the conventional procedure described in [77]. λ-Carrageenan is commercial product of Sigma-Aldrich (Steinheim, Germany).

The sulfated fucooligosaccharides 4F2S(4S), 4F2,3,4S(6S), 6F2S(6S), 8F2S(8S), and 10F2S(10S) were obtained according to the method described in [21], and oligosaccharides 4F2,3S(6S), 6F2,3S(6S), and 8F2,3S(12S) were obtained according to the method described in [27].

### 3.2. Analytical Procedures 

The total carbohydrate amount was determined by the phenol–sulphuric acid method with L-fucose as the standard [78].

The monosaccharide composition after polysaccharides hydrolysis with 2 M TFA (6 h, 100 °C) was determined by using a high-performance anion-exchange chromatography with pulsed amperometric detection (HPAEC-PAD) on an Agilent 1260 Infinity II (Agilent Technologies, Santa Clara, CA, USA) system with electrochemical detector DECADE Elite (Antec Scientific, Zoeterwoude, the Netherlands) using parameters described in [79].

The amount of sulfate groups in carbohydrate samples was determined using the BaCl_2_-gelatin method [80]. 

Protein concentration was determined by the Bradford method [81], with bovine serum albumin as the standard. DNA concentration was determined using micro-spectrophotometer Nano-300 (Allsheng, Hangzhou, China). Protein purity and molecular weight were estimated by SDS-PAGE according to the Laemmli protocol [82]. Electrophoresis was performed in 12% acrylamide gels, with addition of sodium dodecyl sulfate (SDS) detergent. A “Precision Plus Protein Standards” molecular weight marker (Bio-Rad, Hercules, CA, USA) with molecular weights of 10–250 kDa was used as the standard. Gel images were obtained using GS-800 densitometer (Bio-Rad, Hercules, CA, USA). Molecular weights of the proteins obtained via SDS-PAGE were calculated using the QuantityOne 4.6.7 program (Bio-Rad, Hercules, CA, USA).

### 3.3. Amino Acid Sequence Analysis

Identification of the fucoidan-degrading cluster in the genome of *W. fucanilytica* CZ1127^T^ (GenBank: GCA_001697185.1) was described previously [18]. The identification of the putative sulfated polysaccharide-degrading cluster (PUL) containing the gene encoding the putative GH168 family enzyme of the marine bacterium *Z. barbeyronii* KMM6746^T^ (GenBank: GCF_018603515.1) was performed similarly (additional information in pages 2–3 and Figure S1 of the Supplementary Materials). 

Glycoside hydrolases family assignment of the *W. fucanilytica* CZ1127^T^ putative fucanase FWf5 (locus tag: AXE80_07365, GenBank: ANW96105.1) was performed by searching the dbCAN3 meta-server [83]. Multiple sequence alignment (MSA) and the percent identity calculations of amino acid sequences of fucanases were performed using Clustal Omega service (EMBL-EBI service, Hinxton, Cambridgeshire, UK)) [84] and the Jalview software (ELIXIR-UK resource, Version 3, Hinxton, Cambridgeshire, UK)) [85]. A signal peptide was predicted using SignalP (Technical University of Denmark, Version 3.0, Lyngby, Denmark) [86]. InterProScanV5 and NCBI Conserved Domain Database (CDD) were used for functional domain search [87].

### 3.4. Analysis of the Putative Active Site of FWf5 

Data on the spatial structures of fucanases FWf5 (UniProt and AlpaFold accession: A0A1B1Y5R0) and FunA (UniProt and AlpaFold accession: A0A1B1Y6G8) were obtained from the AlphaFold database [88]. Visualization of the protein models and structural analysis of the fucanases’ putative active sites were carried out using PyMol (The PyMOL Molecular Graphics System, Version 1.8, Schrodinger, LLC, New York, NY, USA).

A selection of solvent-accessible amino acid residues within 12Å of D206 in the FunA and D196 in the FWf5 models, respectively, was made using PyMol to identify the amino acid residues of the putative active sites of the enzymes. To evaluate the degree of conservation amino acid residues among both closely and distantly related FWf5 homologs, we selected sets of amino acid sequences that shared between 95–65%, 50–35%, and 45–25% identity with the FWf5 sequence using the ConSurf database [89]. Next, we calculated the conservation of amino acid residues within the proposed FWf5’s active site for each set of amino acid sequences having homologies of 95–65%, 50–35%, and 45–25% using the ConSurf server. The results of the calculations were then mapped onto the FWf5 3D model using PyMol. This provides insight into the conservation of amino acids at the FWf5’s putative active site based on their homology proximity.

### 3.5. Construction and Cloning of the Expression Vectors

The constructs containing the gene encoding Q25-K389 region of FWf5 (GenBank: ANW96105.1), C20-K408 of FunA (GenBank: ANW96599.1), and Q33-H407 of ZbF1 (GenBank: WP_214611692.1) were designed to harbor the C-terminal polyhistidine tag (6×His, vector encoded). The constructs were cloned using restriction-free (RF) cloning strategy [90]. The HF Phusion polymerase (New England Biolabs, Ipswich, MA, USA) was used for RF application. Genomic DNA of *W. fucanilytica* CZ1127^T^ and *Z. barbeyronii* KMM 6746^T^ were used as the templates for amplification of the GH168 family genes. Primer design and polymerase chain reaction (PCR) conditions were carried out using a service at http://www.rf-cloning.org/ (accessed on 1 April 2023). Isolation and purification of genomic DNA were performed using diaGene bacterial genomic DNA kit (diaGene, Moscow, Russia) according to the manufacturer’s protocol. The primers used for cloning genes *fwf5*, *funa*, and *zbf1* are shown in Table 3. The FWf5, FunA, and ZbF1 genes were amplified in PCR with a high-fidelity polymerase (New England Biolabs, Ipswich, MA, USA). The resulting megaprimer was further annealed to the 5207–5297 region of the pet-22b(+) vector (Novagen, Madison, WI, USA) to provide the pet-22b/*fwf5*,pet-22b/*funa,* and pet-22b/*zbf1* recombinant plasmids. After RF cloning, the parental pet-22b(+) vector was eliminated by treating the reaction mixture with the DpnI restriction enzyme (1 μL of 20 U/L for 2 h at 37 °C (New England Biolabs, Ipswich, MA, USA)). Then, the XL10-Gold ultra-competent cells (Agilent Technologies, Santa Clara, CA, USA) were transformed by the RF products. Colony PCR screening was performed using the T7 promoter and the T7 terminator primers. Positive clones were confirmed by DNA sequencing. Isolation of plasmid DNA was performed using Plasmid Miniprep Kit (Evrogen, Moscow, Russia). 

### 3.6. Production of the Recombinant Fucanases of the GH168 Family

Transgenic *E. coli* cell strains of Arctic Express (DE3) (Agilent Technologies, Santa Clara, CA, USA) harboring the pet-22b(+)/*fwf5*, pet-22b(+)/*funa*, or pet-22b(+)/*zbf1* recombinant plasmid were cultivated on lysogeny broth (LB medium) with ampicillin (final concentration 100 μg/mL) in a shaking incubator at 210 rpm and 31 °C for 16 h. Suspension cultures were inoculated in Terrific broth with glucose and lactose (DIAEM, Moscow, Russia) for auto-induction of expression (1:100 *v*/*v*) containing ampicillin (final concentration 100 μg/mL). Recombinant bacterial growth in Terrific broth proceeded at 210 rpm and 31 °C until OD600 = 0.6–0.8, then temperature was lowered to 18 °C, and the growth continued until the cultures’ densities reached saturation (for 24–48 h).

### 3.7. Purification of the Recombinant Fucanases

Every purification step was performed at 4 °C. The bacterial cell cultures were centrifuged at 6000× *g* for 20 min. The obtained bacterial cells were suspended in 0.04 M Tris-HCl (with addition of 0.2 M NaCl and 25 mM of imidazole) pH 7.0 buffer at a ratio of 1:10 (*w*/*v*), then disrupted with sonication at 20 kHz 7 times for 3 min at +1–+8 °C. The suspension was centrifuged at 12,000× *g* for 30 min at +4 °C to remove the cellular debris. The supernatant was subjected to Ni-affinity chromatography on a HisTrap HP cartridge (GE Healthcare, Chicago, IL, USA) equilibrated with 0.04 M Tris-HCl buffer, pH 7.5, that contained 0.2 M NaCl and 25 mM imidazole. The his-tagged proteins were eluted with linear 5–300 mM gradient of imidazole in 0.04 M Tris-HCl buffer, pH 7.0, with addition of 0.2 M NaCl in 50 mL volume at 1 mL/min flow rate. Protein-containing fractions were analyzed for protein bands with the expected molecular weight by sodium dodecyl sulfate–polyacrylamide gel electrophoresis (SDS-PAGE). Fractions containing the target proteins were pooled, concentrated on Vivaspin 10 K devices (Sartorius, Epsom, UK), desalted on a HiTrap desalting column (5 mL, Cytiva, Washington, DC, USA), and equilibrated with 0.04 M Tris-HCl buffer with a pH of 6.8. The resulting fractions were concentrated on Vivaspin 10 K devices (Sartorius AG, Waldbronn, Germany) and mixed with 80% glycerol to the final concentration of 20%. They were stored at −20 °C.

### 3.8. Fucanase Activity Assay

Fucoidanase activity was detected via charged oligosaccharide band occurrence in the gel. The reaction mixture containing 5 μL of enzyme solution (0.01–0.1 mg/mL) in 0.04 M Tris-HCl buffer, pH 6.8 with 100 mM NaCl, and 5 μL of sulfated polysaccharide solution (20 mg/mL) or sulfated oligosaccharide solution (1 mg/mL) in water was incubated at 35 °C for 30 min—24 h. The reaction was stopped by heating at 80 °C for 5 min. Activities of the fucanases were monitored by carbohydrate-polyacrylamide gel electrophoresis (C-PAGE) as described in [18].

Fucoidan FeF isolated from the brown alga *F. evanescens* was used as a substrate for study of FWf5’s biochemical properties. Fucoidan FeF was previously shown to consist mainly of repeating disaccharide units: [→3)Fuc2S-α(1→4)Fuc2S-α(1→] and [→3) Fuc2,4S-α(1→4)Fuc2S-α(1→] [59]. According to SEC analysis, the molecular weight of FeF is about 185 kDa.

### 3.9. pH Optimum Determination for the Activities of Fucanases

The reaction mixtures containing 10 μL of fucanase solution (0.01 mg/mL) in 0.04 M Tris-HCl buffer (pH 6.8) with 5 mM CaCl_2_, 5 μL of buffers with various pH values (0.3 M Na-succinate buffers with pH range of 3.7–6.5; 0.3 M Tris-HCl buffers with pH values from 6.5 to 8.5 or 0.2 M Na-borate buffer, pH 9.0), and 5 μL of the *F. evanescens* fucoidan solution in water (40 mg/mL) were incubated for 1 h at 35 °C. The reaction was stopped by freezing. Activity levels were monitored by C-PAGE as described above.

### 3.10. Influence of Multivalent Metal Ions on the Activities of FWf5

The reaction mixtures containing FWf5 solution (10 μL with 0.01 mg/mL concentration) in 0.04 M Tris-HCl buffer (pH 6.8), 10 μL of the *F. evanescens* fucoidan solution in water (20 mg/mL), and solution of an appropriate salt (2 μL of 0.1 M AlCl_3_, BaCl_2_, CaCl_2_, CuSO_4_, FeCl_3_, MgCl_2_, CoCl_2_, MnCl_2_, NiSO_4_, and SnCl_2_ or 2 μL of 2M NaCl or KCl) were incubated for 1 h at 35 °C. The reaction was stopped by freezing. Activity levels were determined by C-PAGE as described above.

### 3.11. Optimum Temperature Determination for the Activities of FWf5

Fucoidanase solutions (10 μL with concentration of 0.01 mg/mL) in 0.04 M Tris-HCl buffer (pH 6.8) with 100 mM NaCl were incubated with 10 μL of the fucoidan solution (10 mg/mL) in water at different temperatures (25, 30, 35, 40, 45, 50, 55, 60, 65, and 70 °C) for 1 h. Enzyme activity was monitored as described above.

### 3.12. Time Course of Fucoidan Degradation by FWf5

Kinetics of enzymatic fucoidan hydrolysis was evaluated by C-PAGE. The reaction mixture containing 100 μL of fucoidanase solution (0.01 mg/mL) in 0.04 M Tris-HCl buffer (pH 7.0) with 100 mM NaCl and 100 μL of fucoidan solution in water (20 mg/mL) was incubated at 35 °C for 10 and 30 min and 1, 4, and 24 h. Then, 10 μL aliquots of the reaction mixture were taken for each period of time for further C-PAGE analysis. The reaction was stopped by heating at 80 °C for 5 min.

### 3.13. Preparation and Isolation of Enzymatic Hydrolysis Products

The fucoidan from *F. evanescens* (0.3 g) was dissolved in 27 mL of 0.04 M Tris-HCl buffer (pH 6.8) with 100 mM NaCl, and 3 mL of FWf5 (0.1 mg/mL) in the same buffer was added. The reaction mixture was incubated at room temperature (about 25 °C) for 72 h and then deproteinized by heating at 80 °C for 10 min, and the precipitate was removed by centrifugation at 12,000× *g* for 20 min. The high-molecular-weight reaction products (HMP_W5) were precipitated with ice-cold ethanol 96% at a ratio of 1:3 (*v*/*v*), and the precipitate was separated by centrifugation at 10,000× *g* for 40 min. The supernatant containing the low-molecular-weight reaction products (LMP_W5) was evaporated under vacuum to remove ethanol and then diluted with distilled water to 1 L. The obtained solution was applied to a column with Q-Sepharose HP (1 × 10 cm) (GE Healthcare, Chicago, IL, USA) equilibrated with water. Oligosaccharides were eluted with a linear gradient from 0 M to 2 M (150 × 150 mL) of NH_4_HCO_3_ solution in water with subsequent linear gradient from 0.5 to 1.5 M (100 × 100 mL) of NaCl. Fraction volume was 1.5 mL, and flow rate was 1 mL/min. Carbohydrate content in fractions was analyzed by the phenol-sulfuric method. Fractions that contained carbohydrates were further analyzed by the C-PAGE method, as described above. Oligosaccharide fractions that had one band in the electropherogram were pooled, desalted by vacuum evaporator or desalted on a HiTrap desalting column (5 mL, Cytiva, Washington, DC, USA), and then freeze-dried. Structures of the obtained oligosaccharides were determined with NMR spectroscopy.

### 3.14. Fluorescent Labeling of Sulfated Fucooligosaccharides

Sulfated fucooligosaccharide labeling with 2-aminobenzamide (2AB, Sigma-Aldrich, St. Louis, MO, USA) was performed according to [91], with some modifications. In brief, 0.3 M 2AB was dissolved in a mixture of dimethyl sulfoxide (DMSO) and 15% glacial acetic acid that contained 1 M NaCNBH_3_ (Sigma-Aldrich, St. Louis, MO, USA). A total of 75 μL of the prepared 2AB reaction mixture was added to 50 nM of pure oligosaccharides and incubated for 2 h at 60 °C. The labeled oligosaccharides were separated from the reducing agent and the 2AB excess by anion-exchange chromatography on a Q-Sepharose HP column (0.5 × 1 cm) using one-step gradient of 2 M or 2.5 M NH_4_HCO_3_. The eluted oligosaccharides were further desalted and concentrated by vacuum evaporator. Separation of 2AB-labeled oligosaccharides from non-reacting ones was performed by the C-PAGE method, as described above. After electrophoresis, the acrylamide gel was placed in a transilluminator (excitation 365 nm), and the fluorescent oligosaccharide bands were cut out. Acrylamide gel pieces containing the 2AB-labeled oligosaccharides were homogenized with a pestle and extracted with water. The obtained samples of fluorescently labeled oligosaccharides were concentrated by vacuum evaporator and freeze-dried.

### 3.15. Determination of the Molecular Weight of Fucoidan and Its Enzymatic Derivatives 

Molecular weights of polysaccharide samples were determined by size-exclusion chromatography (SEC) using an Agilent 1100 Series HPLC instrument (Agilent, Germany) equipped with a refractive index detector and the series-connected SEC columns Shodex OHpak SB-805 HQ and OHpak SB-803 HQ (Showa Denko, Japan). Elution was performed with 0.15 M NaCl aqueous solution at 40 °C, with a flow rate of 0.4 mL/min. Molecular weight (Mw), number-average molecular weight (Mn), molecular weight of the maximum of the chromatographic peak (Mp), and polydispersity index (PDI) of fucoidans and their derivatives were estimated using standard dextrans of 5, 10, 25, 50, 80, 250, 410, and 670 kDa (Sigma-Aldrich, Steinheim, Germany) as the reference standards.

### 3.16. Determination of the Degree of Polymerization of Oligosaccharides 

Degree of polymerization (DP) of oligosaccharides in the LMP_W5 fraction and during enzymatic hydrolysis of the hexa- (6F2S(6S) and octasaccharides (8F2S(8S) were determined by SEC using an Agilent 1100 Series HPLC instrument (Agilent, Germany) equipped with a chromatography column (15 mm × 500 mm) filled with Superdex 30 prep grade (GE Healthcare Bio-Science, Uppsala, Sweden). Elution of oligosaccharides was performed with 0.2 M NH_4_HCO_3_ solution at RT (24–25 °C) with a flow rate of 0.4 mL/min. Elution of the oligosaccharides was monitored with a refractive index detector. DP distribution of the sulfated fucooligosaccharides in the LMP_W5 fractions was estimated using 2-O-sulfated tetra-, hexa-, octa- and deca-fucooligosaccharides (4F2S(4S), 6F2S(6S), 8F2S(8S), and 10F2S(10S)) as the standards.

Alternatively, the DP’s of oligosaccharides in the LMP_W5 fraction were determined by C-PAGE according to the electrophoretic mobility (Rf) of oligosaccharide bands on acrylamide gel. Oligosaccharides 4F2S(4S), 6F2S(6S), 8F2S(8S), and 10F2S(10S) were used as standards. The electrophoretic mobility (Rf) of the standard oligosaccharides and each oligosaccharide band in the LMP_W5 fraction was calculated using ImageJ software version 1.52a [92]. The Rf values for standards with different DPs were used to construct a calibration curve and calculate the relationship between DP and Rf.

### 3.17. NMR Spectroscopy

One-dimensional (^1^H, ^13^C, TOCSY) and two-dimensional (COSY, ROESY, HSQC, TOCSY) NMR spectra were recorded using an Avance III-700 NMR spectrometer (Bruker Biospin AG, Switzerland) and an Avance II-500 HD spectrometer (Bruker, Germany). Polysaccharides (10–20 mg) or oligosaccharides (2–4 mg) were dissolved in 550 μL of D_2_O, with 1 μL of acetone added as the internal standard (^1^H: 2.225 ppm and ^13^C: 31.45 ppm), and the solution was transferred to a 5 mm NMR tube. The sample tube was inserted in the magnet and allowed to reach thermal equilibrium before performing the experiment. The experiments were run at 35 °C. 

### 3.18. Anticoagulant Activity of the Polysaccharide FeF and Its Enzymatic Derivatives

Activated partial thromboplastin time (aPTT) and prothrombin time (PT) were measured on an APG4-02-P hemostasis analyzer (“EMCO” LLC, Moscow, Russia) using standard reagents “APTT test” and “Renamplastin” (SPD Renam, Moscow, Russia) according to the manufacturer’s recommendations. 

aPTT assay: A solution (15 μL) containing 0.75–15 μg of the fucoidan samples or low-molecular-weightheparin—Clexane (enoxaparin) (“Pharmstandard-UfaVITA” JSC, Ufa, Russia)—in saline solution (0.9% NaCl) was added to 135 μL of the normal human platelet-depleted citrated plasma. The resulting mixture was poured into two measuring cuvettes of 50 μL each. It was then mixed with the same volume of APTT, incubated at 37 °C for 3 min, and placed in the cuvettes. The test result was provided as the average time from two measurement channels from the moment 50 μL of 0.025 M calcium chloride solution was added to the test plasma until clot formation.

PT assay: To the normal human platelet-depleted citrated plasma of 135 µL, 15 µL of a sample of different concentrations (0.75–15 μg) dissolved in saline solution (0.9% NaCl) was added. The mixture was transferred to two measuring cuvettes of 50 µL each, incubated at 37 °C for 2 min, and placed in the measuring cells. The test result was displayed as the average of the time taken from the addition of 100 µL of the PT reagent to the test plasma to the formation of a clot.

TT assay: Thrombin time (TT) was measured in the same manner. Briefly, normal human platelet-depleted citrated plasma (225 μL) was mixed with a solution of 25 μL of sulfated polysaccharides of different concentrations in saline solution (0.9% NaCl). The mixture was then poured into two measuring cuvettes of 100 µL each, incubated at 37 °C for 2 min, and placed in the measuring cells. The measurement started from the moment when 100 µL of stabilized thrombin with an activity of 9 U/mL was added to the test plasma until the moment of clot formation.

The low-molecular-weight heparin (Clexane) was used as a positive control to compare anticoagulant activity. The samples’ anticoagulant activity was assessed using a saline solution as control. All procedures were performed in a minimum of four replicates (n = 4). The results are presented as mean ± S.D. Statistical significance was determined with Student’s *t* test. The *p* values less than 0.05 were considered significant.

## 4. Conclusions

In the present work, we have carried out the identification, bioinformatic analysis, and determination of the biochemical properties of the new enzyme FWf5 of the GH168 family of the marine bacterium *W. fucanilytica* CZ1127^T^. The enzyme was found to catalyze the hydrolysis of 1→4-glycosidic linkages exclusively between 2-O-sulfated L-fucose residues in fucoidans with a backbone consisting of alternating 1→3- and 1→4-linked sulfated α-L-fucose residues, while fucoidans with alternative backbone structures were resistant to the enzyme. We classified this enzyme as the 2-O-sulfated (1→3;1→4)-α-L-fucan endo-1→4-α-L-fucanase (EC 3.2.1.212). To our knowledge, this is the first report on members of the GH168 family capable of catalyzing 1→4-glycosidic linkages between sulfated α-L-fucose residues. The data obtained indicate that the fucanases of the GH168 family, similar to the fucanases of the GH107 family, have different specificities with respect to both the type of glycosidic bond and the pattern of substrate sulfation.

Carbohydrate-modifying enzymes can be used to precisely modify biologically active glycan molecules. Such modifications could potentially alter their biological effects. In this way, enzymes can be used to manipulate the biological properties of glycans. The possibility of using the studied fucanase FWf5 of the GH168 family and recently discovered endo-4-O-sulfatase SWF5 for targeted modification of the structure of the fucoidan FeF from *F. evanescens* was demonstrated. The specificity of the FWf5 for the cleavage of glycosidic bonds within 2-O-sulfated fucoidan fragments made it possible to obtain derivatives with either regular alternating 2-O- and 2,4-di-O-sulfation or regular 2-O-sulfation. Some derivatives were further, specifically 4-O-desulfated by SWF5 sulfatase. The resulting derivatives were evaluated for a comparative study of their anticoagulant activity.

We have shown that some specific modifications of the fucoidan FeF from *F. evanescens* using enzymes can radically change its anticoagulant properties. This allowed us to identify some structural elements of (1→3;1→4)-fucoidans that influence their anticoagulant properties. As shown, the high anticoagulant activity of the FeF fucoidan and its derivatives are primarily related to their molecular weight and the 2,4-di-O-sulfation. Thus, precise manipulation of these structural parameters of the fucoidan FeF can be used to produce the fucoidan derivatives with desired anticoagulant properties.

## Figures and Tables

**Table 1:** ^1^H and ^13^C chemical shifts (ppm) of the HMP_W5.

Residues	Chemical Shifts, ppm.					
	H1/C1	H2/C2	H3/C3	H4/C4	H5/C5	H6/C6
→4-α-L-Fucp2S-1→	5.38/98.4	4.45/76.5	4.37/67.8	4.0/83.1	4.38/68.9	1.36/16.2
→3-α-L-Fucp2,4S-1→	5.37/99.2	4.56/75.2	4.31/73.8	4.95/80.3	4.48/68.0	1.31/16.3

**Table 2:** ^1^H and ^13^C chemical shifts (ppm) of the oligo-fr4 and oligo-fr5.

Residues	Chemical Shifts, ppm.					
	H1/C1	H2/C2	H3/C3	H4/C4	H5/C5	H6/C6
α-L-Fucp2S-(1→3)-	5.36/94.1	4.45/75.8	4.10/68.0	3.90/72.6	4.49/67.3	1.23/15.9
→3)-α-L-Fucp2S-(1→4)-	5.28/99.8	4.57/74.0	4.19/72.6	4.12/69.1	4.40/67.7	1.25/16.0
→4)-α-L-Fucp2S-(1→3)-	5.35/94.2	4.48/75.8	4.16/68.0	3.99/83.3	4.55/67.9	1.37/16.1
→3)-α-L-Fucp2S	5.47/90.9	4.51/74.0	4.06/72.6	4.09/69.1	4.23/66.9	1.25/16.0

**Table 3:** The list of primers used for cloning the genes encoding the enzymes of the GH168 family FWf5 and FunA from *W. fucanilytica* CZ1127^T^ and ZbF1 from *Z. barbeyronii* KMM 6746^T^.

Enzyme Name	Primer Sequences, 5′-3′ (F—Forward Primer; R—Reverse Primer)
FWf5	F: AATTTTGTTTAACTTTAAGAAGGAGATATACATATGCAAGTAAGTTATAAAAATAGTGATGGAAGT R: CTTGTCGACGGAGCTCGAATTTTATTTCCAAATTATTTTAGCCTTTCTTGT
FunA	F: AATTTTGTTTAACTTTAAGAAGGAGATATACATATGTGTAGTACAACAAAAACACACACC R: CTTGTCGACGGAGCTCGAATTTTACTTTTTCCATTCAATTTTCGCTTC
ZbF1	F: AATTTTGTTTAACTTTAAGAAGGAGATATACATATGCAACCATCATTACTTACAAGTGATG R: CTTGTCGACGGAGCTCGAATTTCAATGCCATGTAATTTTCGCC

The underlined and the non-underlined sequences are the vector-specific primers and the gene-specific primers, respectively.

## Data Availability

The data supporting the findings of this study are available within the article and Supplementary Materials.

## Supplementary Materials

The supporting information can be downloaded at https://www.mdpi.com/article/10.3390/ijms25010218/s1, references [93,94,95,96,97] are cited in Supplementary Materials.
