# Supplementary material for: The Discovery of the Fucoidan-Active Endo-1→4-α-L-Fucanase of the GH168 Family, Which Produces Fucoidan Derivatives with Regular Sulfation and Anticoagulant Activity
Source: Int J Mol Sci. 2023 Dec 22;25(1):218. doi: 10.3390/ijms25010218 (PMC10778895; doi:10.3390/ijms25010218)
Supplement: Supplementary file 1 [file ijms-25-00218-s001.zip › ijms-2765659-supplementary.pdf]

Supplementary materials for

Article

The Discovery of the Fucoidan-Active Endo-1→4-α-L-fucanase of the GH168 Family, Which Produces Fucoidan Derivatives with Regular Sulfation and Anticoagulant Activity

Artem S. Silchenko [1,*], Ilya V. Taran [1], Roza V. Usoltseva [1], Nikolay V. Zvyagintsev [2], Anastasiya O. Zueva [1], Nikita K. Rubtsov [1], Dana E. Lembikova [1], Olga I. Nedashkovskaya [3], Mikhail I. Kusaykin [1], Marina P. Isaeva [4] and Svetlana P. Ermakova [1,*]

[1] Laboratory of Enzyme Chemistry, G.B. Elyakov Pacific Institute of Bioorganic Chemistry, Far-Eastern Branch of the Russian Academy of Sciences, 159, Prospect 100-Let Vladivostoku, 690022 Vladivostok, Russia; usoltseva-r@yandex.ru (R.V.U.); zstasya95@gmail.com (A.O.Z.); rubtsov.nk@yandex.ru (N.K.R.); dlembikova@gmail.com (D.E.L.); mik@piboc.dvo.ru (M.I.K.)

[2] Laboratory of Physical and Chemical Research Methods, G.B. Elyakov Pacific Institute of Bioorganic Chemistry, Far-Eastern Branch of the Russian Academy of Sciences, 159, Prospect 100-Let Vladivostoku, 690022 Vladivostok, Russia

[3] Laboratory of Microbiology, G.B. Elyakov Pacific Institute of Bioorganic Chemistry, Far-Eastern Branch of the Russian Academy of Sciences, 159, Prospect 100-Let Vladivostoku, 690022 Vladivostok, Russia; olganedashkovska@piboc.dvo.ru

[4] Laboratory of Marine Biochemistry, G.B. Elyakov Pacific Institute of Bioorganic Chemistry, Far-Eastern Branch of the Russian Academy of Sciences, 159, Prospect 100-Let Vladivostoku, 690022 Vladivostok, Russia; issaeva@gmail.com

Identification of a sulfated polysaccharide degrading cluster in the marine bacterium *Z. barbeyronii* KMM 6746^T

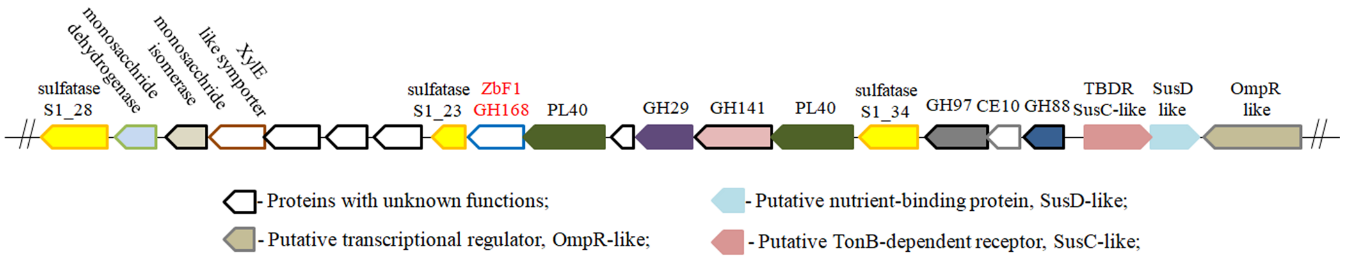

Sulfatase subfamily (SulfAtlas)	Known activities in SulfAtlas	Notes
S1_28	Not known	Associated with carrgenans degradation #1. Associated with fucose-containig sulfated polysacchrides degradation #2
S1_23	Not known	Associated with fucose-containig sulfated polysacchrides degradation #2
S1_34	Not known	Associated with fucose-containig sulfated polysacchrides degradation #2

Glycoside hydrolase (GH) or polysacchride lyase (PL) family (CAZy)	Known activities in CAZy	Notes
GH168	Endo-α-(1,3)-L-fucanase	Associated with fucose-containing sulfated polysacchrides degradation #2-4
PL40	Ulvan lyase	Can be associated with ulvans degradation #5
GH29	α-L-Fucosidase; α-L-glucosidase; *α-L-galactosidase* #6	Frequently associated with a fucoidan degradation#2,3. α-L-Galactosidase activity was detected in the enzyme BpGH29 of the bacterium *Bacteroides plebeius* #6
GH141	α-L-Fucosidase; xylanase	Frequently associated with fucoidan degradation #3
GH97	Glucoamylase; α-L-glucosidase; α-L-galactosidase; β-L-arabinopyranosidase	-
GH88	d-4,5-Unsaturated β-glucuronyl hydrolase; unsaturated chondroitin disaccharide hydrolase; gellan tetrasaccharide unsaturated glucuronosyl hydrolase	Can be associated with ulvan degradation #5

Figure S1. Putative gene cluster involved in the degradation of sulfated polysaccharides in marine bacterium *Z. barbeyronii* KMM 6746^T. #1 – Reference [93], #2 – reference [94], #3 – reference [13], #4 – reference [20], #5 – reference [95], #6 – reference [96].

The genome of *Z. barbeyronii* KMM 6746^T (GenBank: GCF_018603515.1) was analyzed for the presence of sequences similar to known fucanases belonging to the 168 family of glycoside hydrolases (GH168, CAZy). The amino acid sequences of FWf5 (GenBank: ANW96105.1) and FunA (GenBank: ANW96599.1) were used to search for homologs in the *Z. barbeyronii* genome. A BLAST search identified the gene encoding the putative GH168 family enzyme. We named this gene as *zbf1* (GenBank: WP_214611692.1). Analysis using the dbCan metaserver confirmed that ZbF1 belongs to the GH168 family. The amino acid sequence identity between the FWf5 and the ZbF1 was 51%, and between FunA and ZbF1 was 48%. Genes in the immediate vicinity to the *zbf1* gene were manually annotated using the InterProScan service, and the CAZy (dbCan3 metaserver) and SulfAtlas databases [83,97]. It was therefore identified a gene cluster containing several genes encoding the S1 family of sulfatases (subfamilies S1_23, S1_28, and S1_34), the

GH29, GH88, GH97, and GH141 families of glycoside hydrolases, and the PL40 family of polysaccharide lyases. It has been reported that some subfamilies of sulfatases S1_23, S1_28, S1_34 as well as GH29, GH141, and GH168 can be involved in the degradation of fucose-containing sulfated polysaccharides in brown algae [94,13]. At the same time, the genes encoding PL40 and GH88 can be associated with the degradation of ulvans, sulfated polysaccharides of green algae [95]. Thus, the composition of the genes contained in this cluster does not allow a clear answer about the specialization of this cluster to fucoidans, ulvans or other sulfated polysaccharides containing fucose, galctose and uronic acids. Further analysis of the functional activity of the enzymes contained in this cluster will allow the specialization of this cluster to be determined.

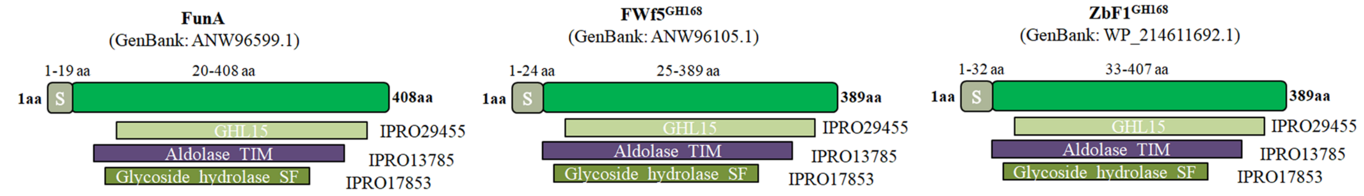

Figure S2. Domain organization of FWf5, ZbF1, and characterized endo-fucanase FunA.

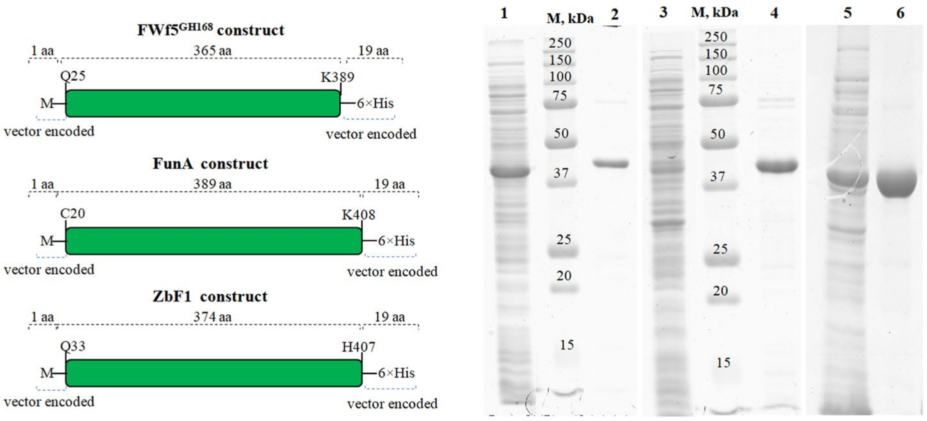

Figure S3. A schematic depicting of the obtained constructs encoding the FWf5 (Q25-K389), FunA (C20-K408), and ZbF1 (Q33-H407) (leftward). SDS-PAGE analysis in 12% polyacrylamide gel of the recombinant *E. coli* cell lysate transformed with the *pet-22/fwf5* (1), the *pet-22/fwf5* (3), and *pet-22/zbf1* (5). SDS-PAGE analysis in 12% polyacrylamide gel of the putative fucanase FWf5 (2), fucanase FunA (4), and ZbF1 (6) after purification on a Ni-sepharose HP column. M - Molecular weight marker.

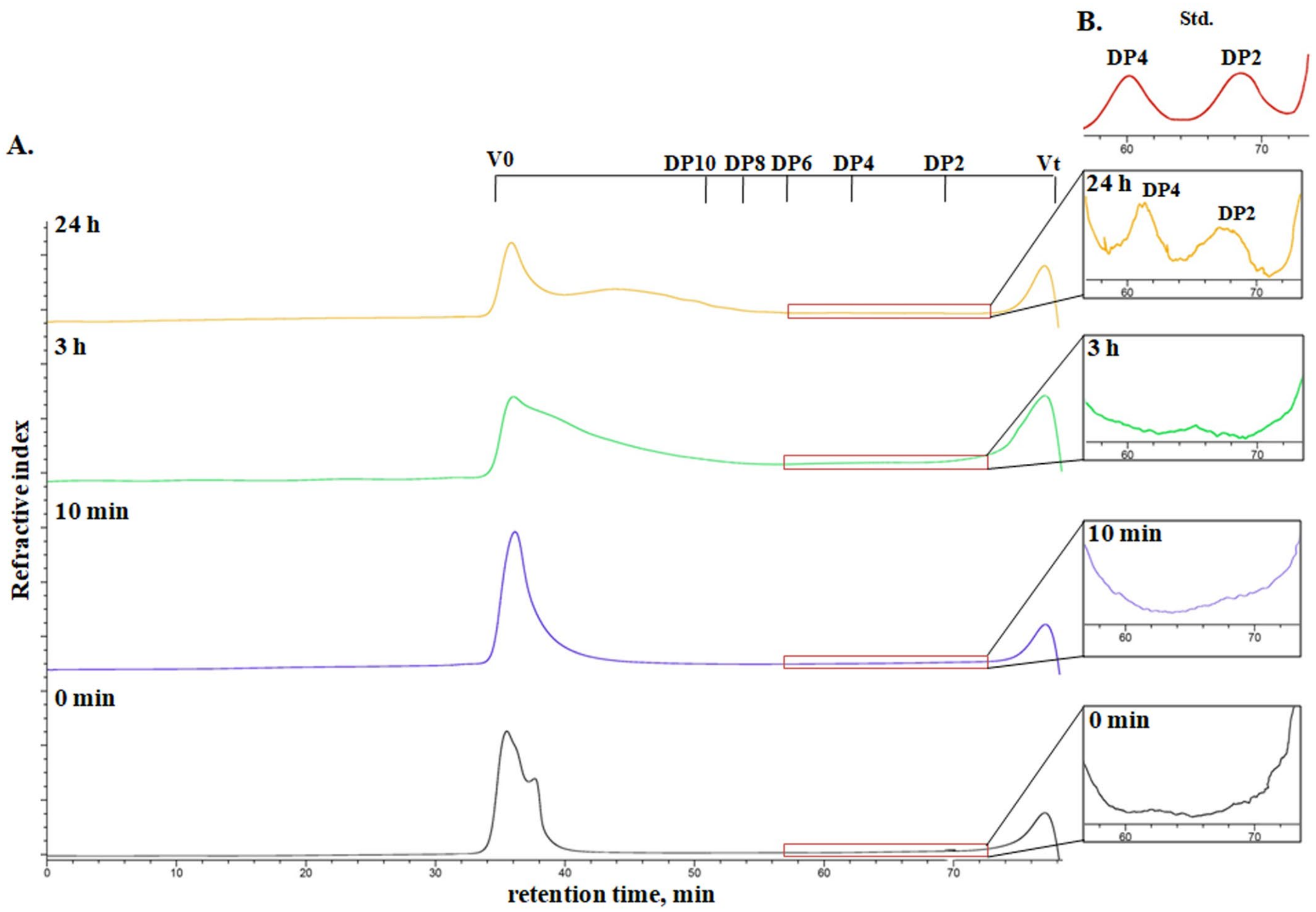

Figure S4. SEC analysis of the hydrolysis products of the fucoidan FeF by the fucanase FWf5 at different incubation times on the Superdex 30 PG column (**A**). (**B**) The relevant part of the chromatograms with increasing scale, showing the retention time area of the di- (DP2) and tetrasaccharides (DP4). Std. - Chromatogram of the oligosaccharides 2F2S(2S) (DP2) and 4F2S(4S) (DP4).

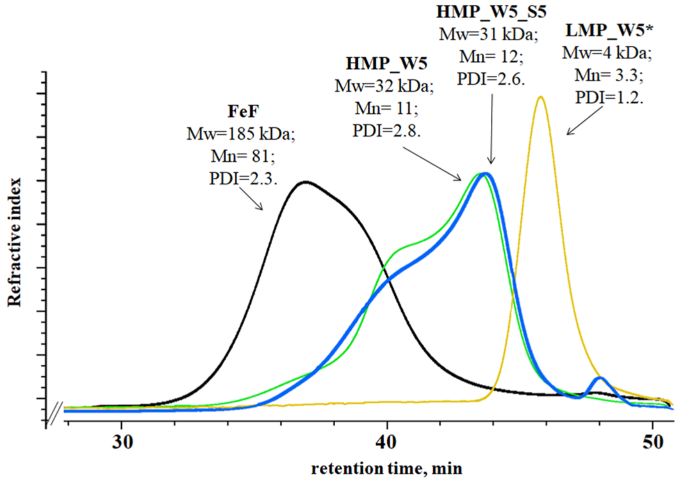

Figure S5. SEC analysis of the fucoidans FeF, HMP_W5, HMP_W5_S5, and LMP_W5.

Table S1. Structural characteristics of fucoidans used in the experiment.

Species of brown alga	Fucoidan or its derivative name	-SO_3Na, %*	Monosaccharide composition, mol. %						
			L-Fuc	L-Rha	D-Gal	D-Glc	D-Man	D-Xyl	UA
F. evanescens	FeF	26	95.9	1.4	1.7	0.4	0.6	0	0
F. vesiculosus	FvF (Sigma-Aldrich)	18	89.9	0	6.9	0	3.2	0	0
S. cichorioides	ScF	30	99.0	0	1.0	0	0	0	0
S. horneri	ShF	21	91.7	0	8.3	0	0	0	0
U. pinnatifida	UpF	23	64.8	0	35.2	0	0	0	0
F. evanescens	LMP-W5 (derivative of the fucoidan FeF)	21	100	0	0	0	0	0	0
F. evanescens	HMP-W5 (derivative of the fucoidan FeF)	30	93.4	2.5	2.3	0.8	1.0	0	0
F. evanescens	HMP-W5_S5 (derivative of the fucoidan FeF)	23	93.5	2.5	2.3	0.7	1.0	0	0

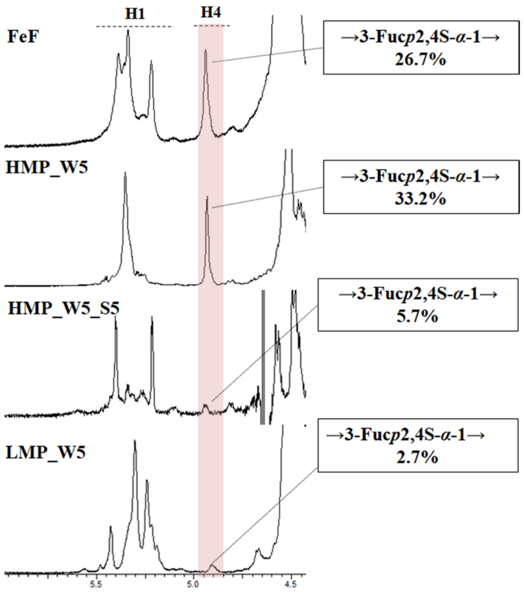

Figure S6. Relevant area of the ^{1}H NMR spectra of the fucoidan FeF and its enzymatic derivatives HMP_W5, HMP_W5_S5 and LMP_W5. The content of the →3-Fuc*p*2,4S-*α*-1→ residues in the samples was calculated as integrated intensities of the all signal of H1 to the signals of H4 of the →3-Fuc*p*2,4S-*α*-1→ residues.

Table S2. Identity matrix of the members of the GH168 family deposited in the CAZy database (August 2023).

	GenBank access	1	2	3	4	5	6	7	8	9	10	11	12	13	14	15	16	17	18	19	20	21	22	23	24	25 FWf5	26	27	28	29	30	31	32	33	34	35	36	37	38	39	40	41	42	43	44	45	46	47	48	49
1	WDE98918.1	100	19.31	19.95	20.16	20.25	20.65	20.25	20.25	20.38	22.79	23.38	21.79	22.35	22.07	21.79	24.11	23.27	22.40	23.83	21.78	23.28	23.94	20.00	21.64	21.41	18.38	20.34	20.67	20.93	21.02	19.51	19.23	22.02	16.96	22.51	20.74	21.41	20.73	20.53	20.81	20.52	20.52	20.23	20.52	21.64	20.06	19.78	21.19	22.06
2	QDT09618.1	19.31	100	19.72	17.91	19.80	19.80	19.80	19.22	19.89	18.88	18.29	18.13	18.70	18.41	18.41	19.60	19.94	17.63	20.05	19.75	18.52	19.35	20.47	21.01	24.19	22.35	22.88	22.95	24.11	21.35	22.84	23.38	20.87	16.41	24.57	25.87	23.48	23.41	24.58	26.18	26.18	26.18	25.59	25.59	25.89	25.88	21.91	27.22	25.66
3	ACI65020.1	19.95	19.72	100	30.29	30.91	30.65	30.65	30.65	27.30	25.00	24.46	24.59	24.59	25.14	24.86	26.91	27.42	27.06	28.16	26.04	28.50	26.51	19.84	22.65	22.82	23.45	22.04	22.99	23.18	21.83	21.56	21.84	24.07	20.51	26.03	23.30	25.55	25.27	27.82	25.07	25.35	25.63	24.79	24.79	25.21	24.17	24.60	25.00	25.07
4	WIH28120.1	20.16	17.91	30.29	100	56.73	56.73	57.05	57.53	28.57	25.32	25.00	25.61	26.16	26.16	26.43	28.22	30.66	27.54	28.97	28.22	22.84	24.35	21.80	21.85	23.69	24.46	23.24	23.77	23.31	23.22	21.24	22.31	22.73	18.10	24.09	22.69	25.69	24.03	27.17	27.45	27.17	26.89	26.33	26.61	27.20	26.87	24.46	27.51	24.52
5	QUM85487.1	20.25	19.80	30.91	56.73	100	99.36	99.36	91.72	27.82	25.65	27.05	27.37	27.91	27.91	27.64	26.92	29.09	28.95	28.79	28.29	23.95	25.71	20.22	19.66	23.48	20.75	21.68	22.74	21.97	22.74	22.91	22.64	23.65	17.58	23.88	22.77	27.70	27.15	27.53	26.12	26.12	26.69	26.12	26.40	25.85	26.39	25.07	26.72	24.59
6	QUM89703.1	20.65	19.80	30.65	56.73	99.36	100	99.36	91.72	28.08	25.65	27.05	27.37	27.91	27.91	27.64	26.92	29.09	28.95	28.79	28.29	23.95	25.96	19.67	19.66	23.48	20.49	21.41	22.47	21.69	22.47	22.64	22.37	23.36	17.00	23.60	22.77	27.15	27.15	26.69	25.84	25.84	26.40	25.84	26.12	25.00	25.56	24.80	26.15	24.31
7	QUM81180.1	20.25	19.80	30.65	57.05	99.36	99.36	100	91.72	28.08	25.65	27.32	27.64	28.18	28.18	27.91	27.20	29.09	28.95	28.53	28.29	23.95	25.96	20.22	19.66	23.48	20.75	21.68	22.74	21.97	22.74	22.91	22.64	23.65	17.58	23.60	23.04	27.42	27.42	27.25	26.40	26.12	26.69	26.12	26.40	25.57	26.11	25.07	26.72	24.59
8	CED59768.1	20.25	19.22	30.65	57.53	91.72	91.72	91.72	100	29.13	25.00	26.78	26.83	27.37	27.37	27.64	26.92	28.25	28.69	29.05	27.80	23.10	24.42	20.77	21.07	22.65	20.75	21.41	22.19	21.97	22.74	22.37	22.37	22.79	17.29	23.88	22.51	25.76	25.48	25.84	25.84	25.84	26.12	25.28	25.56	24.43	26.39	23.43	26.15	23.76
9	ANW96381.1	20.38	19.89	27.30	28.57	27.82	28.08	28.08	29.13	100	54.74	53.32	53.16	53.67	53.67	53.42	35.64	37.56	35.43	38.62	39.14	43.25	44.25	22.55	26.78	27.17	25.81	25.75	24.80	24.17	24.12	24.21	24.80	22.56	23.96	28.31	24.69	28.80	28.27	28.18	26.15	25.88	26.42	26.15	26.42	28.03	25.81	23.48	27.90	26.22
10	ANW96384.1	22.79	18.88	25.00	25.32	25.65	25.65	25.65	25.00	54.74	100	71.65	73.25	72.04	72.34	72.34	42.63	44.55	40.99	44.83	45.34	47.20	50.00	22.52	22.77	26.78	26.44	24.24	23.00	24.67	23.43	25.57	27.21	25.57	24.27	28.57	29.52	29.77	30.42	27.80	28.12	27.48	27.80	27.16	27.80	28.43	27.56	27.16	28.85	28.43
11	QXP78593.1	23.38	18.29	24.46	25.00	27.05	27.05	27.32	26.78	53.32	71.65	100	78.17	78.17	78.93	78.93	44.56	39.95	42.08	42.78	43.90	45.48	48.45	21.85	23.73	26.96	25.14	24.50	26.04	25.94	23.46	26.78	24.86	28.77	22.91	27.06	27.37	28.69	29.22	28.26	27.84	27.57	27.84	27.57	27.30	27.22	27.15	24.67	27.62	28.38
12	SDS23585.1	21.79	18.13	24.59	25.61	27.37	27.37	27.64	26.83	53.16	73.25	78.17	100	97.48	97.98	97.98	41.69	40.89	40.72	42.71	43.30	44.87	47.30	22.91	24.79	27.59	25.21	25.71	25.55	26.57	23.40	26.02	24.39	28.21	23.74	25.73	25.85	29.49	30.56	27.72	27.57	28.11	27.30	27.57	28.11	27.76	28.23	24.40	28.73	27.30
13	QXP65556.1	22.35	18.70	24.59	26.16	27.91	27.91	28.18	27.37	53.67	72.04	78.17	97.48	100	98.99	98.99	41.95	41.15	41.24	42.71	43.81	45.38	47.04	23.46	25.63	27.59	25.76	25.99	26.37	26.57	23.68	26.29	24.66	28.21	24.30	26.53	26.37	29.49	30.83	27.72	26.76	27.30	26.49	26.76	27.30	28.30	28.23	24.14	29.28	26.76
14	QXP63046.1	22.07	18.41	25.14	26.16	27.91	27.91	28.18	27.37	53.67	72.34	78.93	97.98	98.99	100	99.50	42.48	40.89	41.49	42.71	43.56	45.13	47.30	23.18	25.07	27.87	25.76	26.27	26.37	26.29	23.68	26.56	24.66	28.21	24.02	26.53	26.11	29.76	31.10	27.99	27.30	27.84	27.03	27.30	27.84	28.03	28.49	24.40	29.01	27.03
15	QXP71075.1	21.79	18.41	24.86	26.43	27.64	27.64	27.91	27.64	53.42	72.34	78.93	97.98	98.99	99.50	100	42.48	40.89	41.75	42.45	43.30	45.13	47.04	23.46	25.63	28.16	26.04	26.55	26.65	26.86	23.96	26.83	24.93	27.65	24.02	26.53	26.37	29.49	30.83	28.26	27.03	27.57	26.76	27.03	27.57	28.03	28.49	24.40	29.01	27.03
16	QDU86693.1	24.11	19.60	26.91	28.22	26.92	26.92	27.20	26.92	35.64	42.63	44.56	41.69	41.95	42.48	42.48	100	44.53	50.88	48.08	50.51	44.70	45.22	20.77	23.14	23.58	22.99	22.88	24.46	23.03	22.13	22.87	25.61	31.55	24.64	32.43	27.75	30.41	31.23	29.78	29.12	28.02	28.02	27.47	27.75	28.57	27.40	27.88	30.99	32.23
17	QGJ68685.1	23.27	19.94	27.42	30.66	29.09	29.09	29.09	28.25	37.56	44.55	39.95	40.89	41.15	40.89	40.89	44.53	100	50.13	54.24	53.44	47.27	44.94	27.17	27.97	29.07	27.45	28.57	27.27	27.51	27.45	27.45	28.69	30.62	27.12	29.87	28.57	31.08	30.81	33.06	32.15	33.51	32.70	33.24	33.79	32.70	31.61	29.60	32.12	31.78
18	QDV66023.1	22.40	17.63	27.06	27.54	28.95	28.95	28.95	28.69	35.43	40.99	42.08	40.72	41.24	41.49	41.75	50.88	50.13	100	60.25	61.59	43.84	44.95	21.41	23.08	24.58	21.93	23.59	23.10	22.78	21.95	22.05	23.64	30.36	23.88	29.97	25.32	31.64	32.63	31.27	28.53	27.99	28.26	27.99	27.99	27.17	26.88	28.01	29.25	30.73
19	QDV69121.1	23.83	20.05	28.16	28.97	28.79	28.79	28.53	29.05	38.62	44.83	42.78	42.71	42.71	42.71	42.45	48.08	54.24	60.25	100	64.69	44.36	44.81	24.24	25.49	27.59	23.98	24.72	26.43	24.36	25.34	25.46	24.47	30.58	27.73	32.45	27.62	30.83	30.85	31.45	29.19	29.46	29.19	29.19	29.73	30.52	29.19	28.27	31.28	31.00
20	QDT07233.1	21.78	19.75	26.04	28.22	28.29	28.29	28.29	27.80	39.14	45.34	43.90	43.30	43.81	43.56	43.30	50.51	53.44	61.59	64.69	100	41.00	45.23	23.45	24.03	23.96	23.68	22.11	24.47	23.18	21.83	22.45	24.04	33.43	24.51	30.85	26.18	32.10	30.91	31.00	27.15	27.69	27.15	26.88	27.15	30.43	27.32	28.20	31.20	29.29
21	QDV26383.1	23.28	18.52	28.50	22.84	23.95	23.95	23.95	23.10	43.25	47.20	45.48	44.87	45.38	45.13	45.13	44.70	47.27	43.84	44.36	41.00	100	48.67	28.14	25.63	28.37	27.25	28.04	27.93	26.40	27.05	26.49	28.24	30.83	26.46	32.17	24.63	30.67	30.03	30.71	31.89	31.35	31.08	30.81	31.35	30.79	28.72	29.74	32.12	30.50
22	WDE97648.1	23.94	19.35	26.51	24.35	25.71	25.96	25.96	24.42	44.25	50.00	48.45	47.30	47.04	47.30	47.04	45.22	44.94	44.95	44.81	45.23	48.67	100	24.25	25.00	27.04	25.81	26.78	26.61	27.30	26.23	27.13	27.13	27.86	22.01	27.96	24.00	28.80	28.80	27.72	29.00	29.54	30.08	30.62	30.89	25.96	26.95	25.33	29.05	28.65
23	WDE98873.1	20.00	20.47	19.84	21.80	20.22	19.67	20.22	20.77	22.55	22.52	21.85	22.91	23.46	23.18	23.46	20.77	27.17	21.41	24.24	23.45	28.14	24.25	100	45.91	46.05	45.99	47.37	48.71	47.87	48.35	49.37	49.11	26.82	23.88	30.68	25.54	29.97	28.57	33.33	32.59	31.20	30.92	31.20	31.48	32.10	33.43	29.70	31.59	32.14
24	CDF78748.1	21.64	21.01	22.65	21.85	19.66	19.66	19.66	21.07	26.78	22.77	23.73	24.79	25.63	25.07	25.63	23.14	27.97	23.08	25.49	24.03	25.63	25.00	45.91	100	51.09	49.19	49.73	49.60	53.08	51.33	50.26	51.59	23.68	22.73	28.41	27.27	28.00	27.43	29.83	28.90	30.59	30.31	29.75	30.03	27.56	30.20	28.53	28.24	32.19
25	**ANW96105.1 . FWf5**	21.41	24.19	22.82	23.69	23.48	23.48	23.48	22.65	27.17	26.78	26.96	27.59	27.59	27.87	28.16	23.58	29.07	24.58	27.59	23.96	28.37	27.04	46.05	51.09	100	66.58	70.91	57.37	56.76	58.79	59.33	56.22	27.16	26.32	30.21	27.67	33.91	33.05	35.36	35.73	34.29	35.16	34.58	34.87	34.21	35.41	35.21	33.73	34.37
26	QDV25809.1	18.38	22.35	23.45	24.46	20.75	20.49	20.75	20.75	25.81	26.44	25.14	25.21	25.76	25.76	26.04	22.99	27.45	21.93	23.98	23.68	27.25	25.81	45.99	49.19	66.58	100	74.88	53.16	55.20	59.02	60.05	58.31	27.54	25.37	30.23	25.52	32.13	30.08	31.92	30.17	30.45	31.84	30.73	31.01	30.77	31.51	30.65	33.92	33.15
27	QDS94220.1	20.34	22.88	22.04	23.24	21.68	21.41	21.68	21.41	25.75	24.24	24.50	25.71	25.99	26.27	26.55	22.88	28.57	23.59	24.72	22.11	28.04	26.78	47.37	49.73	70.91	74.88	100	55.90	58.40	60.00	59.85	56.42	27.99	25.00	29.60	26.18	32.77	30.58	32.76	30.31	31.44	31.44	31.73	32.01	31.21	33.43	30.77	32.56	31.87
28	**ANW96599.1.**	20.67	22.95	22.99	23.77	22.74	22.47	22.74	22.19	24.80	23.00	26.04	25.55	26.37	26.37	26.65	24.46	27.27	23.10	26.43	24.47	27.93	26.61	48.71	49.60	57.37	53.16	55.90	100	70.23	59.54	60.55	61.04	27.57	23.67	30.25	27.56	30.28	30.94	33.05	31.55	32.68	33.24	32.68	32.68	30.23	32.59	30.79	32.75	33.70

	FunA																																																	
29	ANW97495.1	20.93	24.11	23.18	23.31	21.97	21.69	21.97	21.97	24.17	24.67	25.94	26.57	26.57	26.29	26.86	23.03	27.51	22.78	24.36	23.18	26.40	27.30	47.87	53.08	56.76	55.20	58.40	70.23	100	63.30	63.06	60.42	27.93	21.30	28.49	29.92	32.27	31.10	31.49	31.40	32.85	32.85	33.14	33.14	29.36	31.20	30.17	30.99	32.36
30	QXP78602.1	21.02	21.35	21.83	23.22	22.74	22.47	22.74	22.74	24.12	23.43	23.46	23.40	23.68	23.68	23.96	22.13	27.45	21.95	25.34	21.83	27.05	26.23	48.35	51.33	58.79	59.02	60.00	59.54	63.30	100	65.58	63.07	27.86	24.70	30.59	25.74	33.80	31.84	33.52	30.92	33.15	34.26	34.54	33.98	31.36	30.58	29.97	31.21	33.43
31	QDV26384.1	19.51	22.84	21.56	21.24	22.91	22.64	22.91	22.37	24.21	25.57	26.78	26.02	26.29	26.56	26.83	22.87	27.45	22.05	25.46	22.45	26.49	27.13	49.37	50.26	59.33	60.05	59.85	60.55	63.06	65.58	100	66.43	29.34	24.71	29.95	26.85	34.06	32.71	32.24	30.05	31.69	30.87	31.42	31.97	28.69	29.19	27.82	33.71	33.24
32	QDS94270.1	19.23	23.38	21.84	22.31	22.64	22.37	22.64	22.37	24.80	27.21	24.86	24.39	24.66	24.66	24.93	25.61	28.69	23.64	24.47	24.04	28.24	27.13	49.11	51.59	56.22	58.31	56.42	61.04	60.42	63.07	66.43	100	28.82	25.00	29.92	27.25	34.15	31.99	33.52	29.86	30.41	31.51	30.68	30.41	29.53	29.19	29.52	34.00	34.85
33	WHT23175.1	22.02	20.87	24.07	22.73	23.65	23.36	23.65	22.79	22.56	25.57	28.77	28.21	28.21	28.21	27.65	31.55	30.62	30.36	30.58	33.43	30.83	27.86	26.82	23.68	27.16	27.54	27.99	27.57	27.93	27.86	29.34	28.82	100	26.10	31.30	30.11	33.80	33.99	29.83	30.64	30.08	28.97	29.53	29.25	32.01	31.46	31.40	30.95	31.28
34	QDU58955.1	16.96	16.41	20.51	18.10	17.58	17.00	17.58	17.29	23.96	24.27	22.91	23.74	24.30	24.02	24.02	24.64	27.12	23.88	27.73	24.51	26.46	22.01	23.88	22.73	26.32	25.37	25.00	23.67	21.30	24.70	24.71	25.00	26.10	100	32.39	26.59	29.33	30.73	30.46	33.14	31.43	30.86	31.14	31.43	32.47	32.29	32.50	33.04	33.14
35	UNE48410.1	22.51	24.57	26.03	24.09	23.88	23.60	23.60	23.88	28.31	28.57	27.06	25.73	26.53	26.53	26.53	32.43	29.87	29.97	32.45	30.85	32.17	27.96	30.68	28.41	30.21	30.23	29.60	30.25	28.49	30.59	29.95	29.92	31.30	32.39	100	35.62	33.96	35.03	41.55	38.87	39.14	39.14	38.87	39.14	40.21	39.89	39.47	41.21	41.35
36	UXX79850.1	20.74	25.87	23.30	22.69	22.77	22.77	23.04	22.51	24.69	29.52	27.37	25.85	26.37	26.11	26.37	27.75	28.57	25.32	27.62	26.18	24.63	24.00	25.54	27.27	27.67	25.52	26.18	27.56	29.92	25.74	26.85	27.25	30.11	26.59	35.62	100	31.59	33.67	40.64	42.29	43.88	42.82	42.82	42.82	44.09	46.07	40.92	43.53	41.71
37	QDV86011.1	21.41	23.48	25.55	25.69	27.70	27.15	27.42	25.76	28.80	29.77	28.69	29.49	29.49	29.76	29.49	30.41	31.08	31.64	30.83	32.10	30.67	28.80	29.97	28.00	33.91	32.13	32.77	30.28	32.27	33.80	34.06	34.15	33.80	29.33	33.96	31.59	100	88.57	43.13	39.57	39.84	39.30	38.77	39.30	39.46	37.70	41.56	42.66	41.71
38	QDV43747.1	20.73	23.41	25.27	24.03	27.15	27.15	27.42	25.48	28.27	30.42	29.22	30.56	30.83	31.10	30.83	31.23	30.81	32.63	30.85	30.91	30.03	28.80	28.57	27.43	33.05	30.08	30.58	30.94	31.10	31.84	32.71	31.99	33.99	30.73	35.03	33.67	88.57	100	43.94	38.24	39.57	38.77	38.24	38.77	39.46	38.52	41.19	42.11	40.05
39	MCP4310320.1	20.53	24.58	27.82	27.17	27.53	26.69	27.25	25.84	28.18	27.80	28.26	27.72	27.72	27.99	28.26	29.78	33.06	31.27	31.45	31.00	30.71	27.72	33.33	29.83	35.36	31.92	32.76	33.05	31.49	33.52	32.24	33.52	29.83	30.46	41.55	40.64	43.13	43.94	100	44.18	42.86	42.33	42.59	42.86	48.24	47.86	49.48	52.75	48.53
40	QXP78589.1	20.81	26.18	25.07	27.45	26.12	25.84	26.40	25.84	26.15	28.12	27.84	27.57	26.76	27.30	27.03	29.12	32.15	28.53	29.19	27.15	31.89	29.00	32.59	28.90	35.73	30.17	30.31	31.55	31.40	30.92	30.05	29.86	30.64	33.14	38.87	42.29	39.57	38.24	44.18	100	81.68	81.94	81.41	81.68	61.56	59.52	52.88	55.34	54.38
41	SDS23738.1	20.52	26.18	25.35	27.17	26.12	25.84	26.12	25.84	25.88	27.48	27.57	28.11	27.30	27.84	27.57	28.02	33.51	27.99	29.46	27.69	31.35	29.54	31.20	30.59	34.29	30.45	31.44	32.68	32.85	33.15	31.69	30.41	30.08	31.43	39.14	43.88	39.84	39.57	42.86	81.68	100	95.03	96.07	96.34	59.68	57.94	52.36	53.97	54.91
42	QXP65559.1	20.52	26.18	25.63	26.89	26.69	26.40	26.69	26.12	26.42	27.80	27.84	27.30	26.49	27.03	26.76	28.02	32.70	28.26	29.19	27.15	31.08	30.08	30.92	30.31	35.16	31.84	31.44	33.24	32.85	34.26	30.87	31.51	28.97	30.86	39.14	42.82	39.30	38.77	42.33	81.94	95.03	100	97.91	97.64	59.68	58.20	51.57	53.70	53.85
43	QXP63049.1	20.23	25.59	24.79	26.33	26.12	25.84	26.12	25.28	26.15	27.16	27.57	27.57	26.76	27.30	27.03	27.47	33.24	27.99	29.19	26.88	30.81	30.62	31.20	29.75	34.58	30.73	31.73	32.68	33.14	34.54	31.42	30.68	29.53	31.14	38.87	42.82	38.77	38.24	42.59	81.41	96.07	97.91	100	99.21	60.22	58.20	51.31	53.97	54.38
44	QXP71078.1	20.52	25.59	24.79	26.61	26.40	26.12	26.40	25.56	26.42	27.80	27.30	28.11	27.30	27.84	27.57	27.75	33.79	27.99	29.73	27.15	31.35	30.89	31.48	30.03	34.87	31.01	32.01	32.68	33.14	33.98	31.97	30.41	29.25	31.43	39.14	42.82	39.30	38.77	42.86	81.68	96.34	97.64	99.21	100	60.22	58.20	51.57	54.25	54.38
45	CDF79633.1	21.64	25.89	25.21	27.20	25.85	25.00	25.57	24.43	28.03	28.43	27.22	27.76	28.30	28.03	28.03	28.57	32.70	27.17	30.52	30.43	30.79	25.96	32.10	27.56	34.21	30.77	31.21	30.23	29.36	31.36	28.69	29.53	32.01	32.47	40.21	44.09	39.46	39.46	48.24	61.56	59.68	59.68	60.22	60.22	100	63.44	58.29	57.53	59.73
46	ANW96379.1	20.06	25.88	24.17	26.87	26.39	25.56	26.11	26.39	25.81	27.56	27.15	28.23	28.23	28.49	28.49	27.40	31.61	26.88	29.19	27.32	28.72	26.95	33.43	30.20	35.41	31.51	33.43	32.59	31.20	30.58	29.19	29.19	31.46	32.29	39.89	46.07	37.70	38.52	47.86	59.52	57.94	58.20	58.20	58.20	63.44	100	56.14	57.58	56.77
47	QEF98541.1	19.78	21.91	24.60	24.46	25.07	24.80	25.07	23.43	23.48	27.16	24.67	24.40	24.14	24.40	24.40	27.88	29.60	28.01	28.27	28.20	29.74	25.33	29.70	28.53	35.21	30.65	30.77	30.79	30.17	29.97	27.82	29.52	31.40	32.50	39.47	40.92	41.56	41.19	49.48	52.88	52.36	51.57	51.31	51.57	58.29	56.14	100	58.08	61.04
48	UBM36933.1	21.19	27.22	25.00	27.51	26.72	26.15	26.72	26.15	27.90	28.85	27.62	28.73	29.28	29.01	29.01	30.99	32.12	29.25	31.28	31.20	32.12	29.05	31.59	28.24	33.73	33.92	32.56	32.75	30.99	31.21	33.71	34.00	30.95	33.04	41.21	43.53	42.66	42.11	52.75	55.34	53.97	53.70	53.97	54.25	57.53	57.58	58.08	100	73.42
49	QDT08277.1	22.06	25.66	25.07	24.52	24.59	24.31	24.59	23.76	26.22	28.43	28.38	27.30	26.76	27.03	27.03	32.23	31.78	30.73	31.00	29.29	30.50	28.65	32.14	32.19	34.37	33.15	31.87	33.70	32.36	33.43	33.24	34.85	31.28	33.14	41.35	41.71	41.71	40.05	48.53	54.38	54.91	53.85	54.38	54.38	59.73	56.77	61.04	73.42	100
